# LL-37_Renalexin hybrid peptide exhibits antimicrobial activity at lower MICs than its counterpart single peptides

**DOI:** 10.1007/s00253-023-12887-5

**Published:** 2024-01-13

**Authors:** Julius Kwesi Narh, Nestor G. Casillas-Vega, Xristo Zarate

**Affiliations:** 1 Facultad de Ciencias Quimicas, Universidad Autonoma de Nuevo Leon, Avenida Universidad s/n, Ciudad Universitaria, 66455 San Nicolas de los Garza, NL Mexico; 2 Departamento de Patologia Clinica, Hospital Universitario Dr. Jose Eleuterio Gonzalez, Universidad Autonoma de Nuevo Leon, 64460 Monterrey, NL Mexico

**Keywords:** LL-37, Renalexin, Hybrid antimicrobial peptide, SmbP, CusF3H+, Fusion proteins, IMAC, Recombinant peptides, Time-killing kinetics

## Abstract

**Abstract:**

An alarming global public health and economic peril has been the emergence of antibiotic resistance resulting from clinically relevant bacteria pathogens, including *Enterococcus faecium*, *Staphylococcus aureus*, *Klebsiella pneumonia*, *Acinetobacter baumannii*, *Pseudomonas aeruginosa*, and *Enterobacter* species constantly exhibiting intrinsic and extrinsic resistance mechanisms against last-resort antibiotics like gentamycin, ciprofloxacin, tetracycline, colistin, and standard ampicillin prescription in clinical practices. The discovery and applications of antimicrobial peptides (AMPs) with antibacterial properties have been considered and proven as alternative antimicrobial agents to antibiotics. In this study, we have designed, produced, and purified a recombinant novel multifunctional hybrid antimicrobial peptide LL-37_Renalexin for the first time via the application of newly designed flexible GS peptide linker coupled with the use of our previously characterized small metal-binding proteins SmbP and CusF3H+ as carrier proteins that allow for an enhanced bacterial expression, using BL21(DE3) and SHuffle T7(DE3) *Escherichia coli* strains, and purification of the hybrid peptide via immobilized metal affinity chromatography. The purified tag-free LL-37_Renalexin hybrid peptide exhibited above 85% reduction in bacteria colony-forming units and broad-spectrum antimicrobial effects against *Staphylococcus aureus*, *Escherichia coli*, Methicillin-resistant *Staphylococcus aureus* (MRSA), and *Klebsiella pneumoniae* bacteria clinical isolates at a lower minimum inhibition concentration level (10–33 μM) as compared to its counterpart single-AMPs LL-37 and Renalexin (50–100 μM).

**Key points:**

• *The hybrid antimicrobial peptide LL-37_Renalexin has been designed using a GS linker.*

• *The peptide was expressed with the carrier proteins SmbP and CusF3H+.*

• *The hybrid peptide shows antibacterial potency against clinical bacterial isolates.*

**Supplementary Information:**

The online version contains supplementary material available at 10.1007/s00253-023-12887-5.

## Introduction

Antibiotic resistance, a globalized public health peril in this new era of modern medicine, prevailed from the pathogenic microbial development of novel resistance mechanisms of genetic and epigenetic origins against the available antibiotics by circumventing the therapeutic actions of these drugs, leading to the failure of antimicrobial drugs curbing the menace of infection (Dar et al. 2017; Soares et al. 2021). The discovery, research, and clinical applications of immuno-peptides known as antimicrobial peptides (AMPs), such as defensins, LL-37, gramicidins D, histatins, Renalexin, and others with antibacterial properties, have been considered and proven as alternative antimicrobial agents to antibiotics (Perez-Perez et al. 2021; Peters et al. 2010; Zhang et al. 2021). Antimicrobial peptides are mostly cationic, amphipathic, and composed of 10–100 amino acid residues. These biological agents have shown novel therapeutic potencies against resistant bacterial pathogens elicited via the disruption of the cell wall and plasma membrane, inducing pore formation, upon the establishment of electrostatic interactions with the negatively charged membrane phospholipids, or inhibiting DNA replication and protein synthesis, thereby exhibiting a broad spectrum of therapeutic activities (Jindal et al. 2015; Ołdak and Zielińska 2017). Many AMPs possess a net charge of +2 to +9, confirming strong polarity to bacterial cell membrane surface structures, thereby conceding antibacterial activity against bacterial pathogens that pose global public health threats (Wei and Zhang 2022). AMPs are known for modulation and orchestration and as indispensable components of the innate immune system functioning as the first line of defense against bacterial attack in eukaryotes and often synthesized as a competitive strategy in prokaryotes to limit and outcompete the cellular growth of competitive microbes (Seyedjavadi et al. 2021). The mechanisms of therapeutic action of AMPs, including antibacterial peptides, have been extensively exploited over the years. Studies in both in vitro, in vivo, and model plasma membranes have confirmed novel and distinct modes of action compared to clinically prescribed antibiotics by extensively provoking plasma membrane incision and permeability, leading to membrane disruption and cell death (Erdem Büyükkiraz and Kesmen 2022; Wei and Zhang 2022).

The recent applications of AMPs as disease control therapeutics, coupled with their growing interest as antimicrobial agents produced via recombinant expression systems, have provided a promising and safer platform for the expression and the clinical applications of AMPs *(*Jindal et al. 2015; Montfort-Gardeazabal et al. 2021; Nuti et al. 2017). This study focused on two antimicrobial peptides, LL-37 and Renalexin. LL−37 is the only α-helix cathelicidin-based AMP with therapeutic action against bacterial, viral, and fungal infections (Kang et al. 2019). The biosynthesis of LL−37 occurs in most immune cells, including the mast cell, neutrophils, mucosal epithelial cell, keratinocytes, adipocytes, and the T and B lymphocytes. Cathelicidins (hCAP18) are distinct mammalian immune proteins that act as precursor molecules that undergo proteolytic cleavage at the C-terminal to release a short peptide with antimicrobial and immune-modulatory activity commonly known as LL-37 (Erdem Büyükkiraz and Kesmen 2022). The antibacterial activity of naturally and recombinantly purified LL−37 against multi-drug resistant (MDR) bacteria pathogens has been elucidated and published *(*Dürr et al. 2006; Erdem Büyükkiraz and Kesmen 2022; Perez-Perez et al. 2021; Scott et al. 2002). Antimicrobial activity of single-peptide LL-37 showed promising results against *Staphylococcus aureus* (69% sensitivity) and *Escherichia coli* (64% sensitivity) at 50 μM peptide concentration (Perez-Perez et al. 2021). A study by Douglas Clark and colleagues in early 1994 led to the discovery of a novel antimicrobial peptide Renalexin (sometimes referred to as Ranalexin), isolated from the skin of the American Bullfrog *Rana catesbeiana*. It contains a single intramolecular disulfide bond between two cysteine amino acids at positions 14 and 20, which forms a heptapeptide ring within the molecule similar to that seen in the antibiotic Polymyxin B. Renalexin is initially synthesized as a precursor peptide with a putative signal sequence and an acidic amino acid–rich region at its N-terminal *(*Aleinein et al. 2013; Clark et al. 1994). Renalexin has shown therapeutic actions against both gram-positive *S. aureus* and gram-negative *E. coli* bacteria at concentrations ranging from 50 μM and above by interacting with phospholipid membrane via electrostatic binding; in cases where the peptide traverses the membrane, it inhibits protein expression which leads to cell death (Dar et al. 2017; Nuti et al. 2017).

Here, we report on an effective and reliable design and expression of soluble hybrid antimicrobial peptide LL-37_Renalexin in *Escherichia coli*. We started with synthetic DNA encoding for the target peptide and using CusF3H+ and SmbP as carrier proteins. The recombinant LL-37_Renalexin gene was cloned into the expression vector pET30a+. The fusion peptides CusF3H+_LL-37_Renalexin and SmbP_LL-37_Renalexin were expressed in *E. coli* with isopropyl β-D-1-thiogalactopyranoside induction under optimized conditions. The recombinant tag-free LL-37_Renalexin was purified via immobilized metal affinity chromatography after being released from the carrier proteins by enterokinase treatment. The in vitro antibacterial activities of the hybrid peptide were ascertained and evaluated.

## Materials and methods

### Reagents, plasmid, enzymes, and bacteria strains

*Escherichia coli* DH5α cells employed for routine plasmid propagation and subcloning experiments were supplied by New England Biolabs (NEB) (Ipswich MA, USA). Protease-deficient bacteria strains *Escherichia coli* BL21(DE3) and *Escherichia coli* SHuffle T7(DE3) also supplied by NEB were used as microbial expression hosts. The plasmid pET30a+ purchased from EMD Biosciences (Darmstadt, Germany) was chosen for the design and construction of plasmid expression vectors. The MEGAquick-spin DNA and plasmid purification kits were bought from iNtRON Biotechnology (Seoul, South Korea). Restriction enzymes *Nde*I and *Xho*I were purchased from NEB. T4 DNA ligase, *Vent*, and *Taq* DNA polymerases used for molecular cloning and amplification were provided from NEB as well. The synthetic DNA encoding for the hybrid peptide and the protease enterokinase were purchased from GenScript Inc. (Centennial, Piscataway, USA). Isopropyl-β-D-1-thiogalactopyranoside (IPTG) used for induction of expression was purchased from A.G. Scientific Inc. (San Diego, CA, USA). Kanamycin employed as a selective marker for transformed cells and as a positive control was supplied by Sigma-Aldrich (Darmstadt, Germany). Standard protein markers used for peptide size characterization were purchased from NEB and Bio Basic Inc. (Amherst, NY, USA). All culture media including Luria-Bertani, Tryptic Soy Broth, bacteriological agar, and Mueller-Hinton agar used for microbial cultivation, expression, enrichment, and antimicrobial activity were provided by Sigma-Aldrich (Darmstadt, Germany), and Legacy Biologicals (Mount Prospect, IL, USA). All clinical bacteria pathogens including *S. aureus* and *E. coli* used for the antimicrobial activities were supplied from the University Hospital, Department of Pathology, Clinical Microbiology Laboratory (UANL, Monterrey, Mexico).

### Design of the hybrid AMP LL-37_Renalexin

**Fig. 1:**
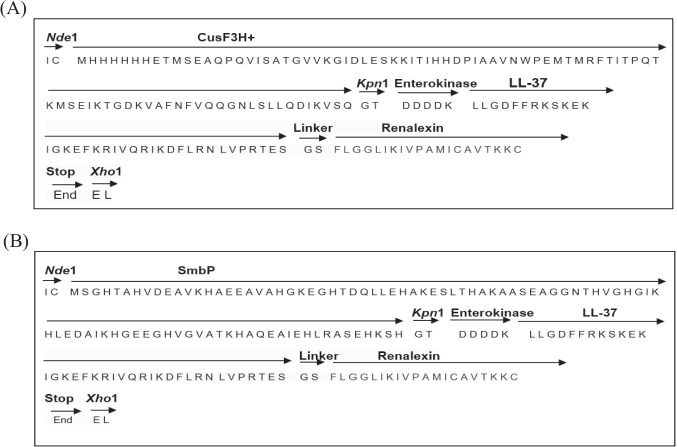
Amino acid sequence flowchart representation of the expression cassette encoding for the recombinant fusion peptides. **A** Expression cassette encoding for CusF3H+_LL-37_Renalexin. **B** Expression cassette encoding for SmbP_LL-37_Renalexin

The mature amino acid sequences of the single antimicrobial peptide LL-37 and Renalexin were retrieved from the AMP database (https://APD3.unmc.edu/structure) with the accession number AP0030/2K60 and AP00513/P39084, respectively, and were used for the design of a novel hybrid peptide LL-37_Renalexin via the application of our newly designed GS peptide linker and the protein tags CusF3H+ and SmbP (Fig. 1) that allow for expression and immobilized metal-affinity chromatography (IMAC) purification. The designed hybrid peptide amino acid sequence was flanked at respective positions with the amino acid sequences of protein tags SmbP or CusF3H+, enterokinase site, GS linker, *Nde*I, *Kpn*I, and *Xho*I restriction enzyme sites. The entire amino acid sequence of the designed complete hybrid peptide was optimized (http://genomes.urv.es/CAIcal) for efficient expression based on codon usage in *E. coli* as the microbial expression host. The biochemical properties and molecular structure of the designed hybrid peptide were elucidated and analyzed using Expasy and I-TASSER tools (https://web.expasy.org/cgi-bin/protparam/protparam, https://zhanggroup.org/I-TASSER/) to access the efficiency and the reliability of production in microbial systems.

### Construction of pET30a+ expression vectors

A 492- and 480-mer oligonucleotide DNA (gene) sequences (Fig. S1) encoding for SmbP_LL-37_Renalexin and CusF3H+_LL-37_Renalexin with the accession numbers OR356112 and OR356113, respectively, (https://www.ncbi.nlm.nih.gov/Genbank.html) were synthesized based on the optimized hybrid peptide sequence with reference to codon usage in *E. coli*. The gene was cloned into pUC57 and supplied to the protein expression and purification laboratory, UANL, Mexico. Both the pUC57 and pET30a+ plasmid vectors were digested with *Nde*I and *Xho*I restriction enzymes (Fig. S2). The restriction digestion products were visualized on a 1% agarose gel stained with 0.5 μg/ml ethidium bromide. The target DNA fragment was cut from the gel and purified using the MEGAquick-spin DNA purification kit. Purified DNA fragments were ligated with the T4 DNA ligase for the design of two plasmid expression vectors pET30a+_CusF3H+_LL-37_Renalexin and pET30a+_SmbP_LL-37_Renalexin. The resulting plasmid construct was transformed for propagation into *E. coli* DH5α by heat shock at 42 °C for 45 s. Transformant cells were selected on Luria-Bertani–kanamycin (30 μg/ml) agar plates. To confirm the presence of the gene in the designed plasmid constructs, the 5′-T7 promoter forward primer (5′-TAATACGACTCACTATAGGG-3′) and the 3′-T7 terminator reserve primer (3′-GCTAGTTATTGCTCACGG-5′) pairs were used for polymerase chain reaction (DNase-free water, 5 mM dNTPs, 10 ng DNA template, 0.5 μM primer, 5U *Taq* polymerase) under the following conditions: initial denaturation at 95 °C for 1 min, denaturation at 95 °C for 30 s, primer annealing at 55 °C for 45 s, elongation at 72 °C for 50 s, and final elongations at 72 °C for 5 min, 32 reaction cycles of amplification. The PCR amplicons were analyzed on a 1% agarose gel and visualized under a UV transilluminator (Fig. S3). The plasmid constructs were sequenced by STARSEQ GmbH (Instituto de Biotecnologia, UNAM, Mexico). FinchTV version 1.4 was employed to analyze and confirm the sequenced nucleotides using EMBOSS (data not shown) (Fig. S4).

### Expression and purification of recombinant fusion peptides CusF3H+_LL-37_Renalexin and SmbP_LL-37_Renalexin

For expression, the designed recombinant plasmid DNA construct was transformed by heat shock at 42 °C for 45 s into *E. coli* BL21(DE3) and *E. coli* SHuffle T7(DE3) calcium competent cells. An inoculum of 5 ml LB broth (30 μg/ml kanamycin) was made, inoculated with a fresh single colony of transformed cells, and incubated at 37 °C overnight with shaking at 220 rpm. Overnight cultures confirming cell viability and plasmid stability were used to inoculate 1000 ml LB broth (30 μg/ml kanamycin) in a baffled flask and incubated at 37 °C, 220 rpm for about 3–4 h until optical cell density (OD_600nm_) of 0.4–0.6 was obtained. Recombinant fusion peptide expression was induced with 1 M isopropyl β-D-1-thiogalactopyranoside (IPTG) at a final concentration of 1 mM. Induced culture flasks were incubated under the optimized condition of 25 °C, 220 rpm for 16 h. Cell pellets harboring the expressed recombinant fusion peptide were harvested by centrifugation at 8500 × g, 4 °C for 15 min into sterilized 50-ml Eppendorf tubes. Collected pellets were resuspended in ice-cold lysis buffer (500 mM NaCl, 50 mM Tris-HCl pH 8.0) and lysed by vortexing on ice with 0.1 mm glass beads. Clear soluble cell lysate was obtained after centrifugation at 8500 × g, 4 °C for 15 min. Purification of recombinant fusion peptide was performed via IMAC using the ÄKTA Prime Plus System (GE Healthcare) for fast protein liquid chromatography (FPLC). Briefly, a 1-ml HisTrap FF agarose-resin Ni(II) charged column was employed for the IMAC purification. The column was equilibrated with 5 column volumes (CV) of equilibrating buffer (500 mM NaCl, 50 mM Tris-HCl pH 8.0). The clarified soluble lysate was loaded onto the column under the conditions of 0.5 MPa pressure and 0.5 ml/min flow rate. Subsequently, the column was washed with 3 CVs of washing buffer (500 mM NaCl, 2.5 mM Imidazole, 50 mM Tris-HCl pH 8.0). The recombinant fusion peptide was eluted by gradient elution with elution buffer (500 mM NaCl, 200 mM Imidazole, 50 mM Tris-HCl pH 8.0). A 10 μl cell lysate, column flow-through, and elution fractions were analyzed on a 15% sodium dodecyl sulfate-polyacrylamide gel electrophoresis (SDS-PAGE), and the purity of protein bands (Tab. S1) was measured by densitometry using ImageJ software (v.2.0) (Adamíková et al. 2019).

### Enterokinase cleavage and purification of LL-37_Renalexin

To induce enterokinase treatment, the purified fusion peptide elution fractions were pooled together in a 6-cm dialysis membrane and dialyzed against dialysis buffer (1× PBS pH 7.2) to desalt the purified peptide and exchange the elution buffer. In brief, the dialysis setup was incubated at room temperature for 1 h with gentle shaking on a magnetic stirrer, followed by overnight incubation at 4 °C. The concentration of the dialyzed fusion peptide was estimated by Bradford analysis using the Bovine Serum Albumin (BSA) (Tab. S2, Fig. S5 and Tab. S3, Fig. S6) as standard protein (Colyer and Walker 1996). A 1 mg purified fusion peptide was exposed to 20 U enterokinase cleavage (5 U/μl) for the release of hybrid peptide LL-37_Renalexin (tag-free). The cleavage reaction was incubated at room temperature for 16 h, followed by enzyme inactivation at −20 °C for 3 h. 10 μl of cleavage mixture was analyzed on a 15% and 18% Tricine SDS-PAGE.

In purifying the tag-free hybrid peptide by IMAC, a 1.0 × 10-cm chromatographic syringe column was loaded with 1 ml of agarose resins charged with 1 M Ni(II) ions. The column was equilibrated with 10 CV of 1× PBS pH 7.2, and the enterokinase cleavage mixture was loaded into the column, mixed, and incubated at 4 °C for 1.5 h for efficient binding of protein tags to the affinity column. Column flow-through was collected as tag-free hybrid AMP LL-37_Renalexin. The protein tags were released with elution buffer (500 mM NaCl, 200 mM Imidazole, 50 mM Tris-HCl pH 8.0). The purified tag-free hybrid peptide was characterized on a 15% Tricine SDS-PAGE, and the concentration was estimated by Bradford analysis and Nanodrop absorbance readings at 280 nm (Tab. S4, Fig. S7 and Tab. S5, Fig S8).

### Antimicrobial activity assay of recombinant hybrid peptide LL-37_Renalexin

#### Culture media

Tryptic soy broth (TSB): tryptone (pancreatic digest of casein) 17.0 g, soytone (peptic digest of soybean) 3.0 g, glucose (dextrose) 2.5 g, sodium chloride 5.0 g, and dipotassium phosphate 2.5 g, pH 7.3. Bacteriological agar: Mueller–Hinton agar (MHA), beef extract 2.0 g, acid hydrolysate of casein 17.5 g, starch 1.5 g, agar 17.0 g, pH 7.3. Phosphate-buffered saline (PBS); sodium chloride 8 g, potassium chloride 0.2 g, sodium phosphate dibasic 1.44 g, potassium phosphate monobasic 0.245 g, pH 7.4. All culture media and buffers were used for microbial cultivation of bacteria clinical isolates, inoculum suspension preparations, and antimicrobial activity tests.

#### Inoculum preparation

Single colonies of test bacteria clinical isolates were cultured in 5 ml TSB and incubated at 37 °C, 220 rpm for 16 h. A 20 μl overnight culture was inoculated into 5 ml TSB and incubated at 37 °C with shaking until an optical cell density (OD_600nm_) of 0.8–1.0 was obtained (mid-logarithmic growth). Cells at the log growth phase were serially diluted in 1× PBS pH 7.2 buffer and the dilution suspensions at 1 × 10^5^ CFU/ml equivalent to 0.5 McFarland turbidity standard were employed for antimicrobial assay.

#### Antimicrobial activity

##### Dose-response assay: minimum inhibition concentration (MIC) determination

The antimicrobial activity of the hybrid peptide was evaluated against gram-positive and gram-negative bacteria clinical isolates of *Staphylococcus aureus*, *Escherichia coli*, methicillin-resistant *Staphylococcus aureus*, and *Klebsiella pneumoniae* from the University Hospital. The minimum inhibition concentrations against the test pathogens were ascertained and estimated in accordance with the modification of the National Committee for Clinical Laboratory Standards (NCCLS) for the broth microdilution method (CLS 2022). Briefly, bacteria cell cultures in the mid-log growth phase in TSB were serially diluted in 1× PBS pH 7.2 buffer to 1 × 10^5^ CFU/ml. A 2-fold broth microdilution assay was performed in a 96-well microtiter plate with a 200 μl assay volume per well composed of 100 μl diluted peptide at concentrations ranging from 0.5–33 μM, 80 μl bacteria suspensions, and 20 μl TSB medium. The plate was incubated at 37°C for 3 h with shaking. After 3 h incubation, 0.1 ml aliquot was taken per well and a 10-fold dilution was made from which a 100 μl aliquot was spread on tryptic soy agar (TSA). Inoculated plates were incubated at 37 °C for 20 h and the remaining colony-forming units were evaluated (Tab. S6, S7, S8, S9, S14, and S15), and MICs were calculated as the lowest peptide concentration that obviate visible turbidity using a modified B. Gompertz function for the line of best fit in dose-response analysis (Lambert and Pearson 2000).

##### Time-killing assay

The antibacterial killing kinetics of the hybrid peptide was evaluated against two bacterial isolates by ascertaining the time course to kill test bacteria isolates suspension of *S. aureus* (gram+), and *E. coli* (gram−). In brief, bacterial cultures in the mid-logarithmic growth phase were incubated as described previously with the peptide LL-37_Renalexin at a concentration of approximately 2× MIC in a TSB medium. The 10 μl aliquot suspensions were taken at every 20 min interval until a period of 3 h incubation was observed. A 10-fold dilution in 1× PBS pH 7.2 was made, and 0.1 ml aliquots were inoculated on TSA medium. Inoculated plates were incubated at 37 °C for 20 h. Log remaining CFU/ml of test pathogens were taken and plotted against time (Tab. S10, and S11). Two control samples were made, positive control (bacterial suspension and kanamycin at the same peptide concentration) and negative control (bacterial suspension and 1X PBS buffer). The average of the total remaining CFU/ml from each treatment was evaluated (Tab. S12 and S13) and analyzed via one-way analysis of variance (ANOVA). The hybrid peptide with a single disulfide linkage (S–S bond) expressed in *E. coli* SHuffle T7(DE3) showing relatively lower minimum inhibitory concentrations (MICs) was employed for time-killing kinetic assay.

## Results

### Design of hybrid peptide and construction of recombinant plasmids

**Fig. 2:**
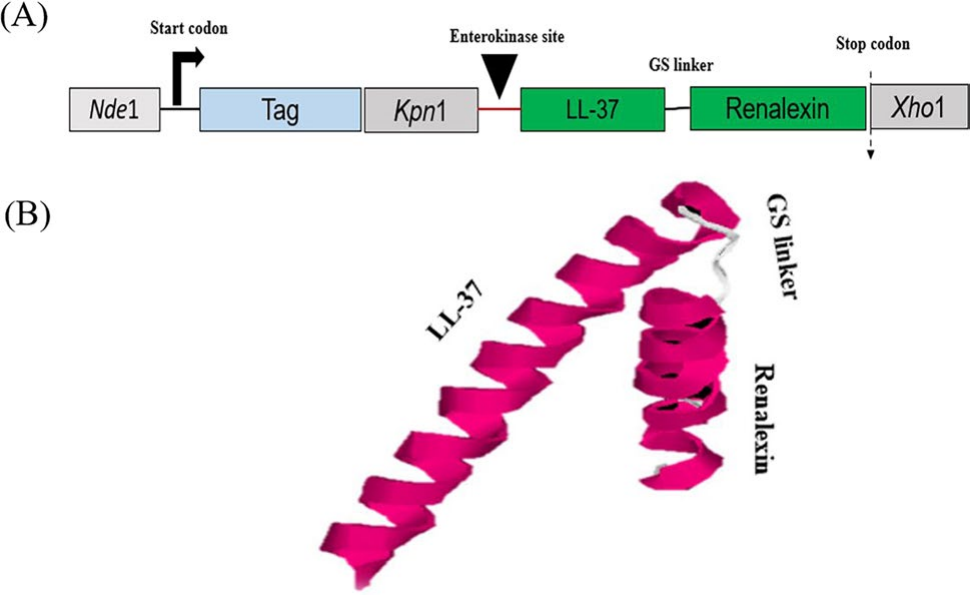
Design of the hybrid antimicrobial peptide LL-37_Renalexin. **A** Representation of expression cassette gene map for LL-37_Renalexin expression in *E. coli*. **B** 3D Riben molecular secondary structure predicted for the hybrid peptide LL-37_Renalexin showing the components including LL-37 (long peptide on left), the flexible GS peptide linker (midway), and Renalexin (short peptide on far right). The peptide structure was predicted in i-Tasser server and confirmed in Phyre2 server with good positive *z*-score (1.00) and *c*-score (−2.09) suggesting an efficient sequence alignment with good, modelled confidence level supporting the predicted structure

In designing the hybrid peptide with the molecular gene map shown (Fig. 2A), we employed the mature amino acid sequences that encode for peptides LL-37 and Renalexin retrieved from the antimicrobial peptide database http://aps.unmc.edu/AP/prediction/ with the accession number AP00310/2K60 and AP00513/P39084, respectively. The amino acid GS (glycine and serine) between LL-37 and Renalexin in the gene construct was employed as a novel, simple, flexible peptide linker allowing for the construction of the hybrid peptide LL-37_Renalexin. DDDDK amino acid sequence at the N-terminal of the hybrid peptide serves as an enterokinase site that allows for the molecular cleavage of the protein tags SmbP and CusF3H+ yielding a tag-free hybrid AMP LL-37_Renalexin. For efficient production and affinity purification, the carrier proteins CusF3H+ and SmbP were inserted between the LL-37_Renalexin gene sequence and the start codon. The molecular Riben structure (Fig. 2B) of the designed hybrid peptide predicted in i-TASSER (https://zhanggroup.org/I-TASSER/) suggests a secondary structural motif with efficient production in *E. coli* (Kesidis et al. 2020). The structure predicted and modeled in i-Tasser was confirmed in the Phyre2 server (Fig. S9) (http://www.sbg.bio.ic.ac.uk/phyre2) with 99.9–99.3% confidence level under the model template identifier (i.d) c2K6oA (LL-37) and c2fcgF (Renalexin) (Kelley et al. 2016). The application of GS peptide linker facilitates the design of a novel hybrid peptide by maintaining the molecular α-helix structure in LL-37 and the heptapeptide ring of Renalexin (https://APD3.unmc.edu/structure). The target hybrid peptide has the following biochemical properties: 59 amino acid length, +9 net charge, pI of 10.3, 44% hydrophobicity and 56% hydrophilicity, GRAVY index of 0.00, instability index of 21.3, aliphatic index of 102.37, and a molecular weight of 6.740 kDa theoretically predicted in http://www.expasy.org/tools/proparameter. The physicochemical properties of the hybrid peptide suggest strong electrostatic cationic polarity, hydropathicity, stability, and good isoelectric potentials which provide theoretical evidence of strong antimicrobial activity of this hybrid peptide at a varying pH range compared to its counterpart single peptides. Cellular toxicity of the hybrid peptide against healthy human cells was ascertained using ToxIBTL advanced bioinformatic online server (https://server.wei-group.net/ToxIBTL/server.html). ToxIBTL is an online in silico peptide and protein toxicity prediction tool that operates using evolutionary information and the physicochemical properties of peptide sequence via the integration of bottleneck principle to predict peptide or protein toxicity level. Our in silico cytotoxicity analysis (Fig. S10) showed that the hybrid peptide has no toxic effect (zero toxicity) on normal human cells at the niche of infection at a toxicity score of 3.7139784e-05 (0.0000371) which is by far below the standard threshold of 0.5.

The DNA nucleotide (gene) of the coding sequence (CDS) was chemically synthesized by GenScript based on the optimized amino acid sequence that encodes for the recombinant fusion proteins CusF3H+_LL-37_Renalexin and SmbP_LL-37_Renalexin. The gene map of the synthetic DNA constituted restriction sites for *Nde*I, *Kpn*I, *Xho*I, and enterokinase, a protein tag site at the N-terminal, with the hybrid peptide site between the protein tag site and a stop codon. The 492-bp (SmbP tag construct) and 480-bp (CusF3H+ tag construct) synthetic DNAs were molecularly cloned into pET30a+ at *Nde*I and *Xho*I restriction sites under the control of T7 promoter using the T4-DNA ligase for the design of two plasmid expression vectors pET30a+_CusF3H+_LL-37_Renalexin and pET30a+_SmbP_LL-37_Renalexin. The plasmid construct was confirmed by colony-based PCR screening using the T7 promoter and terminator-specific primers. After colony-based PCR screening, the correct recombinant plasmid sequence was further confirmed by DNA sequencing (data not shown).

### Expression and IMAC purification of recombinant fusion protein

**Fig. 3:**
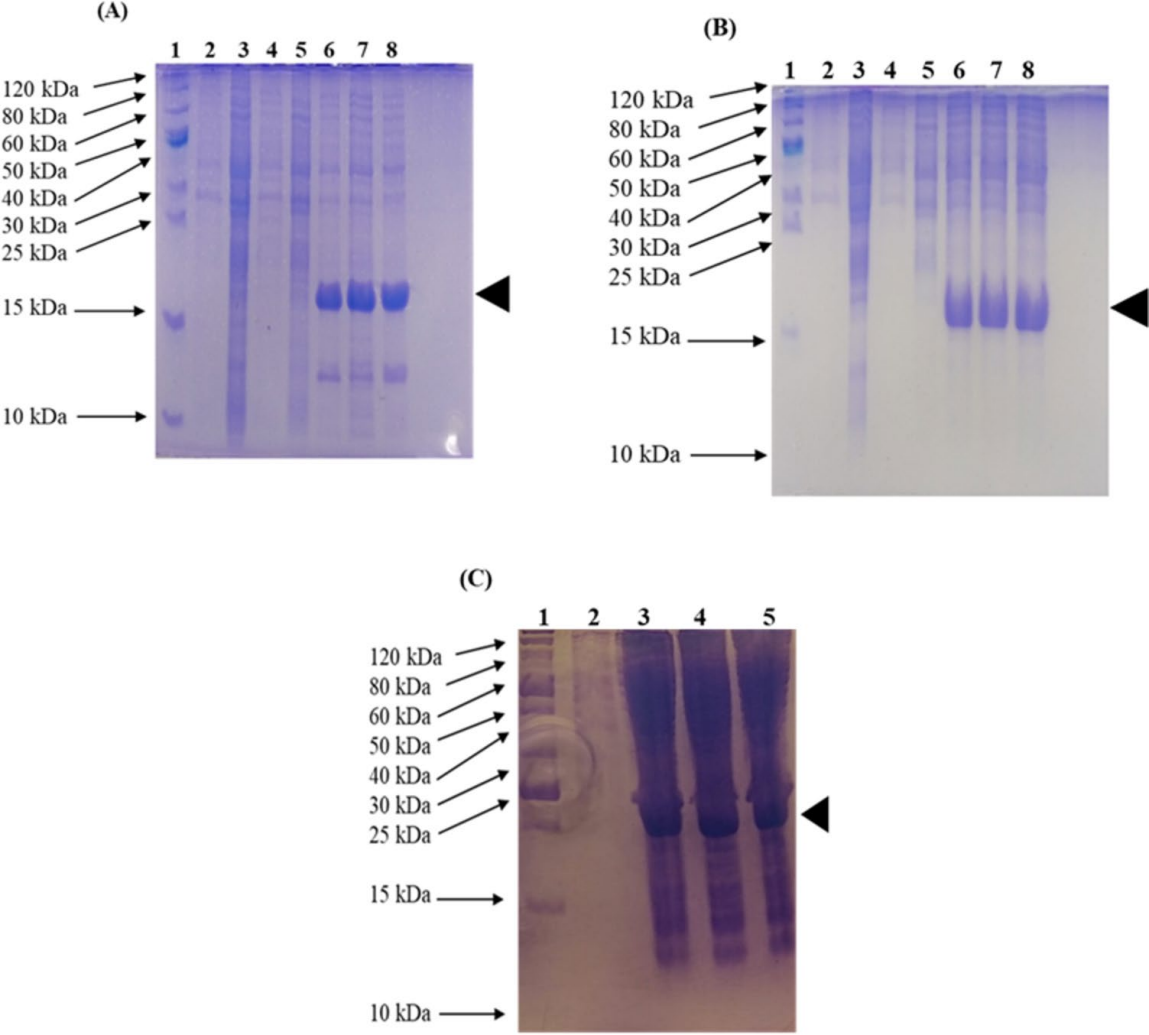
Small-scale expression of recombinant fusion peptides in *E. coli*. **A** 15% SDS-PAGE analysis of CusF3H+_LL-37_Renalexin expressed in *E. coli* BL21(DE3): Lane 1—protein ladder; Lanes 2 and 3—soluble (SF) and insoluble (IF) fractions of untransformed control cells; Lanes 4 and 5—SF and IF of uninduced transformed cells; Lanes 6, 7, and 8—SF of induced transformed cells. **B** 15% SDS PAGE analysis of SmbP_LL-37_Renalexin expressed in *E. coli* BL21(DE3): Lane 1—protein ladder; Lanes 2 and 3—SF and IF of untransformed control cells; Lanes 4 and 5—SF and IF of uninduced transformed cells; Lane 6, 7, and 8—SF of induced transformed cells. **C** 15% SDS-PAGE analysis of CusF3H+_LL-37_Renalexin expressed in *E. coli* SHuffle T7(DE3): Lane 1—protein ladder; Lane 2—soluble fraction of uninduced transformed cells (control); Lanes 3, 4, and 5—SF of induced transformed cells

The protease-deficient bacterial strains *E. coli* BL21(DE3) and *E. coli* SHuffle T7(DE3) competent cells used as expression hosts encode for the T7 RNA polymerase allowing for the expression of the recombinant fusion peptides CusF3H+_LL-37_Renalexin and SmbP_LL-37_Renalexin under the influence of T7 promoter. IPTG induction and expression of fusion peptides successfully showed an efficient insertion of DNA under the T7 promoter in the designed plasmid constructs mentioned above. The carrier proteins CusF3H+ and SmbP show significant advantages in the production and purification of the recombinant fusion peptides expressed as soluble proteins with no formation of inclusion bodies (Perez-Perez et al. 2021; Vargas-Cortez et al. 2016). In evaluating small-scale expression of recombinant fusion proteins CusF3H+_LL-37_Renalexin and SmbP_LL-37_Renalexin (17 kDa) in *E. coli* BL21(DE3) and *E. coli* SHuffle T7(DE3), soluble cell lysates were prepared by lysing cell pellets collected from 2 ml of 1 mM IPTG-induced overnight cultures to access the presence of the target protein in soluble cell lysate. Analysis of 5 μl aliquot of clear lysate on a 15% SDS-PAGE showed successful evidence of expression of the target fusion proteins with 17–18 kDa-expected protein band compared to non-induced cells as negative control samples (Fig. 3). For the large-scale production of recombinant fusion proteins, *E. coli* strains *E. coli* BL21(DE3) and *E. coli* SHuffle T7(DE3) were used as expression hosts. From a 1-L expression volume, cell pellets were collected from 1 mM IPTG-induced overnight cultures and lysed by mechanical vortexing on ice with 0.1 mm glass beads. After cell lysis, clear soluble lysate was collected and employed as a protein source for the immobilized metal affinity chromatography.

**Fig. 4:**
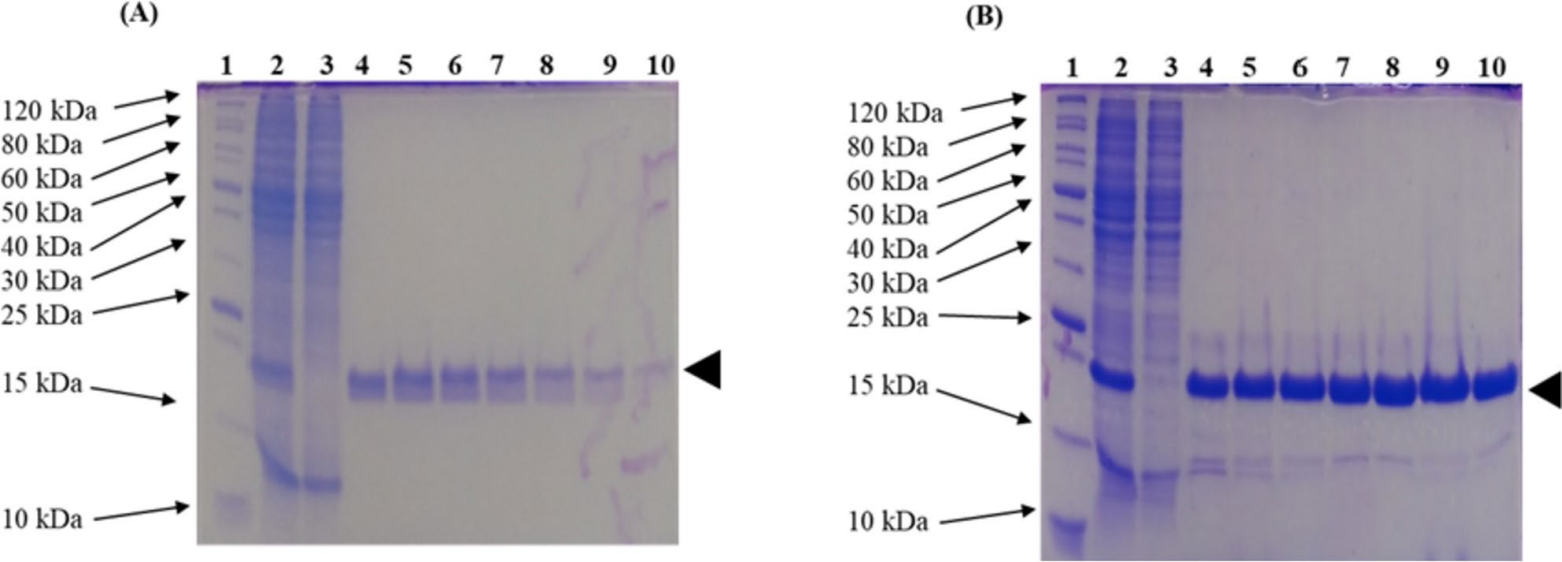
Large-scale expression and IMAC purification of recombinant fusion peptides. **A** IMAC purification of SmbP_LL-37_Renalexin (17 kDa) expressed in *E. coli* BL21(DE3). A 15% SDS-PAGE analysis of elution fractions. Lane 1, protein marker; Lane 2, cell lysate; Lane 3, column flow-through; Lane 4–10, elution fractions. **B** IMAC purification of CusF3H+_LL-37_Renalexin (17 kDa) expressed in *E. coli* SHuffle T7. A 15% SDS PAGE analysis of the elution fractions. Lane 1, protein marker; Lane 2, cell lysate; Lane 3, column flow-through; Lane 4-10, elution fractions

For the purification of fusion protein SmbP_LL-37_Renalexin and CusF3H+_LL-37_Renalexin, the ÄKTA Prime Plus system (GE Healthcare Systems) was employed for fast protein liquid chromatography (FPLC) by metal affinity chromatography. A 1-ml HisTrap FF column charged with Ni(II) was used to isolate the target recombinant peptide from the pool of cellular proteins present in the soluble lysate fractions collected. Analysis of IMAC purification fractions of the recombinant fusion protein on a 15% SDS PAGE (Fig. 4) showed evidence of target peptide in the elution fractions (200 mM imidazole) without any trace of peptide indications in the column flow-through. This result confirms the high affinity of CusF3H+ and SmbP to agarose-resin Ni(II)-charged column that facilitates the binding of the target proteins unto the column while the untargeted cellular proteins exit the column as flow-through (Montfort-Gardeazabal et al. 2021; Perez-Perez et al. 2021; Vargas-Cortez et al. 2017). Our Bradford quantification analysis using standard Bovine Serum Albumin (BSA) calibration equations (data not shown) indicated a peptide concentration of 3.136 mg/L for CusF3H+_LL-37_Renalexin produced in *E. coli* SHuffle T7(DE3) and 1.523 mg/L for SmbP_LL-37_Renalexin produced in *E. coli* BL21(DE3) with a purity of 90–95% matching the purity standard of commercially available synthetic therapeutic peptides. We observed a 2-fold higher recombinant fusion peptide yield in *E. coli* SHuffle T7(DE3) compared to production in *E. coli* BL21(DE3).

### Enterokinase cleavage and purification recombinant LL-37_Renalexin

**Fig. 5:**
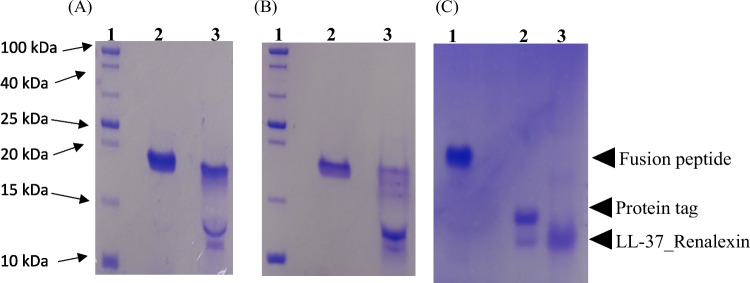
Enterokinase cleavage, tag removal, and second IMAC purification of tag-free LL-37_Renalexin analyzed on Tricine SDS-PAGE. **A** 18% Tricine gel—Lane 1, protein ladder; Lane 2, CusF3H+_LL-37_Renalexin (uncut) expressed in *E. coli* SHuffle T7(DE3); Lane 3, cut CusF3H+_LL-37_Renalexin (enterokinase mix). **B** 18% Tricine gel—Lane 1, protein ladder; Lane 2, SmbP_LL-37_Renalexin (uncut) expressed in *E. coli* BL21(DE3); Lane 3, cut SmbP_LL-37_Renalexin (enterokinase mix). **C** 15% Tricine gel—Lane 1, fusion peptide CusF3H+_LL-37_Renalexin (uncut); Lane 2, enerokinase mix (Protein tag and tag-free LL-37_Renalexin); Lane 3, second IMAC purified hybrid peptide LL-37_Renlexin (tag-free)

Our previous studies (Montfort-Gardeazabal et al. 2021; Perez-Perez et al. 2021) reported the abrogative effect on the bioactivity of recombinant antimicrobial peptides with attached protein tags. We employed the restriction enzyme enterokinase for the selective cleavage of protein tags CusF3H+ and SmbP from the above-purified fusion peptides. Electrophoretic analysis of inactivated enterokinase cleavage mixture revealed three protein bands of size 18 kDa (uncleaved fusion peptide), ≈13 kDa (CusF3H+ or SmbP), and ≈10 kDa (LL-37_Renalexin, tag-free) on Tricine SDS-PAGE (Fig. 5). The uncleaved fusion peptide detected was due to an incomplete enzymatic cleavage reaction. Finally, the tag-free LL-37_Renalexin was purified via a one-step IMAC purification using an agarose resin Ni(II)-charged syringe column unto which the uncleaved fusion peptide and the carrier proteins CusF3H+ and SmbP remain bounded, allowing for the elution of the target hybrid peptide LL-37_Renalexin as column flowthrough. A 2.16 mg/L for tag-free LL-37_Renalexin expressed in *E. coli* SHuffle T7(DE3) and 0.72 mg/L expressed in *E. coli* BL21(DE3) were obtained after Bradford and Nanodrop spectrometry (A280nm) quantification analysis.

### Antimicrobial activity of recombinant LL-37_Renalexin

**Fig. 6:**
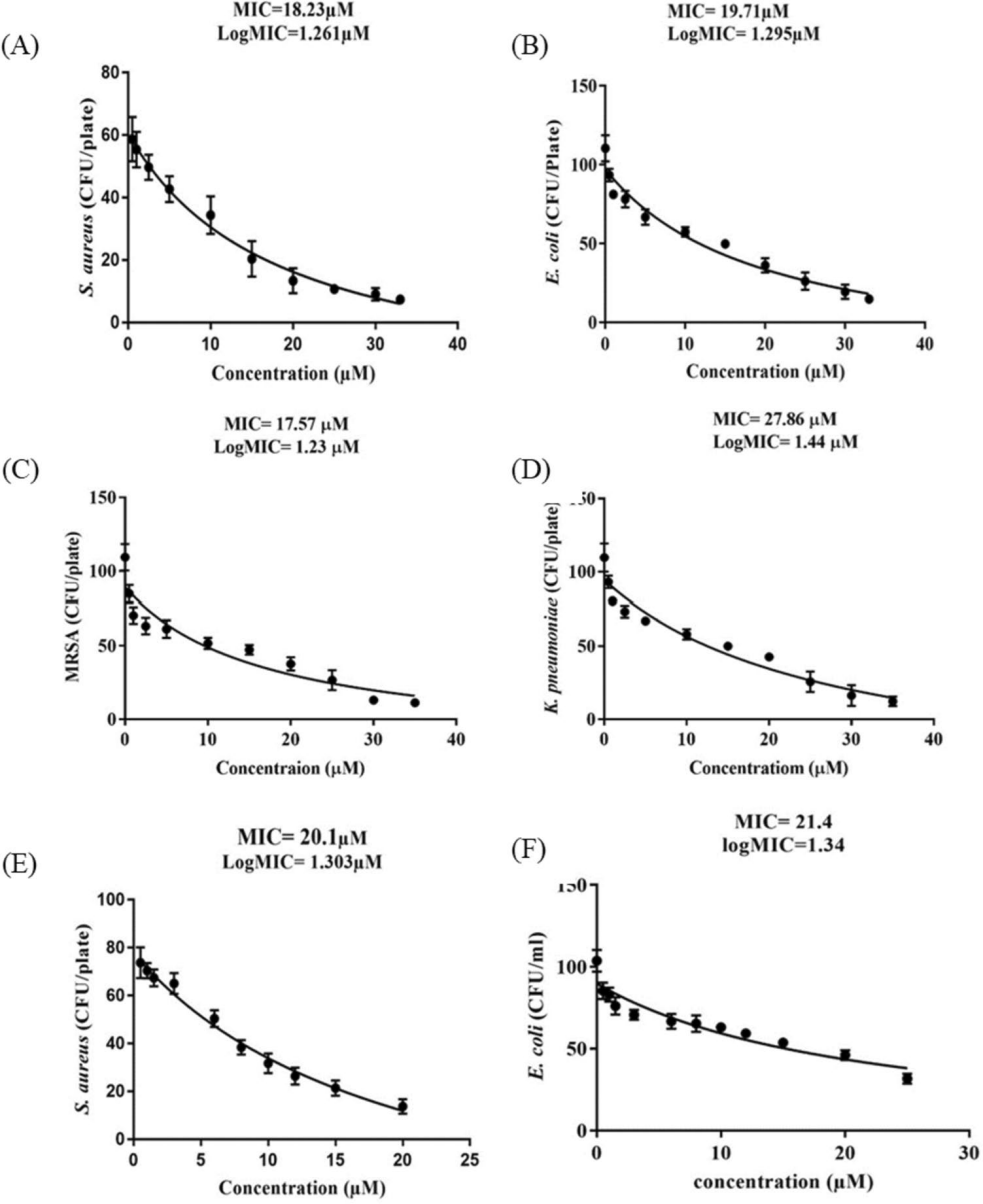
Antimicrobial activity of purified recombinant hybrid AMP LL-37_Renalexin (tag-free) against 1 × 10^5^ CFU/ml of bacteria pathogens. **A** Dose-response activity of LL-37_Renalexin expressed in *E. coli* SHuffle T7(DE3) against CFU/ml of *S. aureus*. **B** Dose-response activity of LL-37_Renalexin expressed in *E. coli* SHuffle T7(DE3) against CFU/ml of *E. coli*. **C** Dose-response activity of LL-37_Renalexin expressed in *E. coli* SHuffle T7(DE3) against CFU/ml of MRSA. **D** Dose-response activity of LL-37_Renalexin expressed in *E. coli* SHuffle T7(DE3) against CFU/ml of *K*. *pneumoniae*. **E** Dose-response activity of LL-37_Renalexin expressed in *E. coli* BL21(DE3) against CFU/ml of *S. aureus*. **F** Dose-response activity of LL-37_Renalexin expressed in *E. coli* BL21(DE3) against CFU/ml of *E. coli*. The data points represent the mean remaining CFU/ml of the test pathogen from three replica plates, and the error bar represents the standard deviation of the mean. Statistical analysis was performed using Gompertz sigmoid function for non-linear regression between the peptide concentration and the CFU/ml of test pathogen

The antimicrobial potency of the purified hybrid peptide against clinical isolates of *S. aureus*, *E. coli*, MRSA, and *K. pneumoniae* was evaluated, and the minimum inhibitory concentrations (MICs) were determined via the broth microdilution assay as described by NCCLSI (Lacy et al. 2004). Data on remaining colony-forming units (CFU/ml) of test pathogens were taken and analyzed after overnight culture with the peptide. The MIC, defined as the minimum peptide concentration that prevented visible turbidity in the test pathogen, was calculated using a modified Benjamin Gompertz sigmoid function (Lambert and Pearson 2000) from the plot of peptide concentrations against the remaining CFU/ml (dose-response plot). The dose-response plots (Fig. 6) show the antibacterial activity of the hybrid AMP LL-37_Renalexin at MIC levels of 10–27 μM, much lower than the reported MICs of the single-peptide LL-37 and Renalexin (50–100 μM) (Aleinein et al. 2013; Kang et al. 2019; Perez-Perez et al. 2021). The MIC result we observed in this study affirms with data reported on related hybrid and dimeric peptides tested against the same bacterial pathogens (Cheng et al. 2021; Dürr et al. 2006; Kang et al. 2019; Wei and Zhang 2022; Seyedjavadi et al. 2021). The dose-response antimicrobial activity results indicated that the test bacterial pathogens were sensitive to the recombinant hybrid peptide at active peptide concentrations as low as 10 μM and 33 μM. Interestingly, we evaluated the bioactivity of the hybrid peptide without disulfide-linkage expressed in *E. coli* BL21(DE3); the results (Table 1) suggested no significant difference in the MICs compared to the hybrid peptide with disulfide-linkage expressed in *E. coli* SHuffle T7(DE3).

**Table 1:** MICs of LL-37_Renalexin expressed in BL21(DE3) and SHuffle T7(DE3) *E. coli* strains

Pathogens	MICs: LL-37_Renalexin (μM)	
	*E. coli* BL21(DE3)	*E. coli* SHuffle T7(DE3)
*Staphylococcus aureus*	20.1	18.2
*Escherichia coli*	21.4	19.7
MRSA	N/A	17.5
*Klebsiella pneumoniae*	N/A	27.8

*N/A* Not Analyzed (peptide expressed in *E. coli* SHuffle T7(DE3) was used for subsequent bioactivity)

### Time-kill kinetics analysis

**Fig. 7:**
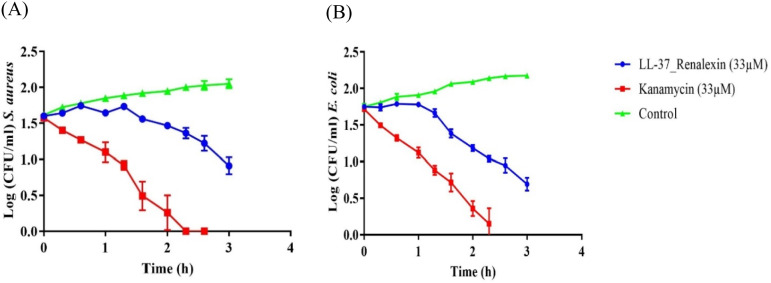
Time-killing kinetics of LL-37_Renalexin (tag-free) expressed in *E. coli* SHuffle T7(DE3) at 2X MIC against the log 1 × 10^5^ CFU/ml of the test pathogens within 3 h time interval of treatment. **A** Time-kill assay of the hybrid peptide against *S. aureus.* **B** Time-kill assay of the hybrid peptide against *E. coli*. A 1 × PBS buffer (pH 7.2) and suspensions of bacteria inoculum were used as the negative control. Antibiotic kanamycin was employed as positive control. The data points represent the mean of log remaining CFU/ml of the test pathogens from three replica plates, and the error bars represent the standard deviation (SD) of the mean CFUs

**Fig. 8:**
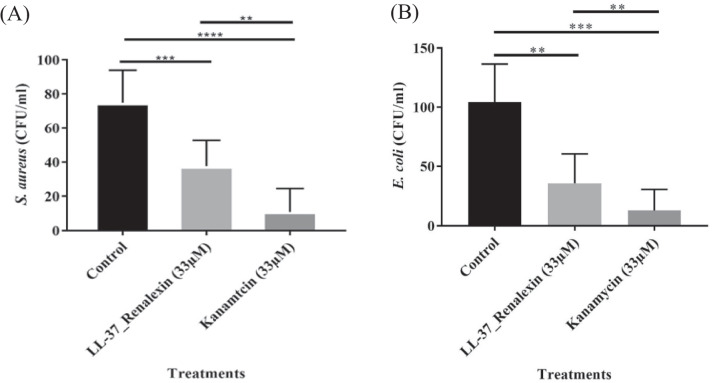
Antimicrobial activity of the hybrid peptide LL-37_Renalexin (tag-free) expressed in *E. coli* SHuffle T7(DE3) against 1 × 10^5^ CFU/ml of test pathogens analyzed by one-way analysis of variance (ANOVA). **A** Antibacterial activity against the CFU/ml of *S. aureus*, **B** Antibacterial activity against the CFU/ml of *E. coli*. A 1 × PBS buffer (pH 7.2) and suspension of bacteria inoculum were used as negative control. The bars represent the mean remaining CFU/ml of the test pathogens from three replica plates and the error bars represent the standard deviation (SD) of the means. Asterisks indicate the statistical significance difference (all *P*-values < 0.05) between the peptide, the negative control, and kanamycin (positive control)

The time-kill kinetic assay (Fig. 7) disclosed that the hybrid peptide shows a multifunctional antibacterial activity within 1.5 h via disruption of membrane integrity and membrane traversing, demonstrating a strong but relatively slow antibacterial potency against all investigated gram-positive and gram-negative bacterial pathogens as compared to the classical antibiotic kanamycin that exhibited its antibacterial activity within less than an hour. We observed that the antibacterial activity of the hybrid peptide in comparison to the known antibiotic kanamycin analyzed against total remaining CFU/ml showed a significant difference at *p*-values < 0.05 (Fig. 8), with the hybrid peptide showing relatively similar antibacterial actions as exhibited by kanamycin (Aleinein et al. 2013; Hanafiah et al. 2020; Montfort-Gardeazabal et al. 2021; Perez-Perez et al. 2021).

## Discussion

Antimicrobial peptides (AMPs) as drug candidates are being considered the new hope for the biomedical and pharmaceutical industries in conjunction with their multifunctional antibacterial pharmacological actions against infectious agents (Montfort-Gardeazabal et al. 2021; Moretta et al. 2021; Nuti et al. 2017). The production of AMPs as therapeutic peptides via the applications of recombinant DNA technology (rDNA) and the use of cost-effective microbial expression systems have facilitated the large-scale acquisition of bioactive AMPs, enhancing clinical and research applications (Akhtar et al. 2012; Dar et al. 2017). In addition to the successful application of rDNA, the efficient, fast, and easy growth of microbial expression hosts like *E. coli* BL21(DE3) and *E. coli* SHuffle T7(DE3) has allowed for commercial production of bioactive cationic AMPs independent of posttranslational modifications (Aleinein et al. 2013; Klubthawee et al. 2020; Montfort-Gardeazabal et al. 2021). Novel AMPs like Brevinin-2R, Renalexin, Cecropin A, and LL-37 as single peptides have been produced as recombinant protein-based drug candidates in microbial systems and have shown a promising antimicrobial effect (Aleinein et al. 2013; Chhetri et al. 2015; Zhang et al. 2018). The thriving commercial production of AMPs in *E. coli* largely depends on the design and use of protein tags, including but not limited to poly-histidine, maltose-binding protein (MBP), thioredoxin protein (THX), and glutathione S-transferase (GST), that allow for the expression of recombinant proteins as fusion proteins in benign forms (Akhtar et al. 2012; El-Gayar 2015; Gddoa Al-sahlany et al. 2020; Mo et al. 2018; Riguero et al. 2020). Recently, our newly designed small metal-binding proteins SmbP and CusF3H+ have been exploited as protein tags which aided in the production and purification of AMPs like LL-37 and Bin1b, and green fluorescence protein (GFP) in different *E. coli* strains (Montfort-Gardeazabal et al. 2021; Perez-Perez et al. 2021; Vargas-Cortez et al. 2016; Vargas-Cortez et al. 2017). Our previous study has unraveled the capabilities of these protein tags that facilitate the secretion, folding, and purification of expressed recombinant LL-37 and Bin1b as single peptides with intact bioactivity at purity above 80% (Montfort-Gardeazabal et al. 2021; Perez-Perez et al. 2021; Santos et al. 2019). Peptide hybridization has been considered an advanced technique for the design of novel AMPs having reliable peptide stability, long half-life with intact therapeutic activity with hybrid peptides like Cecropin A_Thanatin, and Indolicidin_Renalexin showing broad-spectrum antimicrobial activity against multidrug-resistant bacterial pathogens (Bayarbat et al. 2016; Seyedjavadi et al. 2021; Wade et al. 2019).

The broad-spectrum antibacterial activity of LL-37 as a single peptide although at a higher peptide concentration has led us to design a recombinant hybrid peptide production strategy via the application of GS flexible peptide linker and a carrier proteins CusF3H+ and SmbP. The simple GS peptide linker enhances the expression of the hybrid peptide LL-37_Renalexin and maintains the spatial configuration within the hybrid peptide with intact and advanced bioactivity. This data strongly suggests that the GS peptide linker can be employed as a reliable, simple, flexible linker for the design and expression of recombinant therapeutic peptides as compared to the RGGPDGSGPDESGPDE flexible linker employed in the design of hybrid and dimeric peptides with primary structural modifications (Klubthawee et al. 2020; Seyedjavadi et al. 2021).

In this study, we have efficiently employed the mature amino acids of LL-37 and Renalexin for the design of a novel hybrid peptide LL-37_Renalexin with zero cytotoxicity against healthy human cells. We reliably cloned the cDNA that encodes for the target peptide into pET30a+ under the T7 promoter and terminator regions and obtained a successful expression in *E. coli* BL21(DE3) and *E. coli* SHuffle T7(DE3) under the condition of 25 °C, for 16 h with 1 mM IPTG. In other studies, the microbial strains, induction, and temperature conditions demonstrated profound effect on the expression of both single, hybrid, and dimeric peptides with inclusion bodies indications (Chhetri et al. 2015; Montfort-Gardeazabal et al. 2021; Seyedjavadi et al. 2021; Shang et al. 2020; Wade et al. 2019; Xu et al. 2014). The expression condition and peptide isolation protocols observed in this study made it possible for efficient production coupled with higher yield.

Recombinant fusion peptides CusF3H+_LL-37_Renalexin and SmbP_LL-37_Renalexin expression level and its presence in soluble cell lysate provide an evidential advantage of protein tag CusF3H+ and SmbP over others like glutathione S-transferase, maltose-binding protein, amyloid-β peptide, and thioredoxin tag (Aleinein et al. 2013; Chhetri et al. 2015; Zhang et al. 2018) yielding up to 95% peptide purity which matched the purity standard of commercially available synthetic therapeutic peptides (Zhongxuan et al. 2020). In this present study, Bradford quantification of purified and PBS-dialyzed protein elution fractions revealed a total recombinant peptide yield of 1.5–3.1 mg/L fusion proteins SmbP_LL-37_Renalexin and CusF3H+_LL-37_Renalexin expressed in BL21(DE3) and SHuffle T7(DE3), respectively. We observed a 2-fold higher peptide yield in *E. coli* SHuffle T7(DE3) for both SmbP and CusF3H+ tagged fusion proteins than in *E. coli* BL21(DE3), providing relevant supportive data on the usage of *E. coli* SHuffle T7(DE3) as microbial host for the production of either single or hybrid recombinant hybrid peptides with or without disulfide bonds (Montfort-Gardeazabal et al. 2021). Our result showed a higher expression level in recombinant hybrid peptide as soluble protein than reported from other studies, 0.9 mg/L by Seyedjavadi et al. (2021); 900 μg/L by Clement et al. (2015), and 0.3 mg/L by Cheng et al. (2021).

We employed the enzyme enterokinase for the selective cleavage of protein tags CusF3H+ and SmbP from the purified fusion peptides due to the presence of an enterokinase site between the hybrid peptide and the protein tag. Analysis of inactivated enterokinase cleavage mixture revealed three protein bands of an approximate molecular size of 18 kDa (uncleaved fusion peptide), 13 kDa (CusF3H+ or SmbP), and 10 kDa (LL-37_Renalexin, tag-free) on Tricine SDS-PAGE with tag-free recombinant hybrid peptide showing slightly higher band size than the theoretically expected size (6.740 kDa). This result can be attributed to the inefficient enterokinase cleavage observed, which may be associated with the presence of phenylalanine, leucine, and isoleucine residues and heptapeptide motif (Rana box) in Renalexin that forms a cyclic disulfide bond aiding a molecular structural loop formation folded unto the N-terminal that is known to influence enzymatic cleavage and peptide reduction. Also, the high hydrophobicity of the peptide which prevents complete reduction with SDS and mercaptoethanol reagents, and the peptide molecular folding in aqueous systems all of which influence poor peptide mobility. These findings agree with previous studies reported where the single peptides LL-37 and Renalexin show higher protein band sizes than the theoretically determined sizes (Aleinein et al. 2013; Perez-Perez et al. 2021). A 0.7–2.1 mg/L LL-37_Renalexin (tag-free) peptide was obtained upon spectroscopic and Bradford quantification of the second IMAC purification elution fractions (Montfort-Gardeazabal et al. 2021; Perez-Perez et al. 2021; Vargas-Cortez et al. 2017).

The relatively slow induction of the antibacterial activity of LL-37_Renalexin compared to the known antibiotic kanamycin we observed in the time-killing kinetic assay results can be related to the high hydrophobicity of the hybrid peptide which may cause partial exposure of hydrophilic regions to bacterial membrane (Wei and Zhang 2022) coupled with the presence of monovalent Na(I) and K(I) cations in the assay medium that are known to cause shielding effects between the cationic peptides and anionic bacterial membrane surface, hence, the delay in eliciting antibacterial activity in the case of the hybrid peptide as compared to kanamycin (Huan et al. 2020; Nuti et al. 2017). Our findings show that the hybrid peptide LL-37_Renalexin with 44% hydrophobicity and 56% hydrophilicity elicited near-microbicidal activity against all tested pathogens with above 85% reduction in bacteria colony-forming units at 33 μM peptide concentration. The reduction in CFU/ml observed suggests a bactericidal activity since the level of reduction (*R*_L_) in CFU/ml is more significant than three times the logarithm CFU/ml of bacteria (Klubthawee et al. 2020; Zhang et al. 2018). All *S. aureus*, *E. coli*, MRSA, and *K. pneumoniae* clinical isolates showed 85% sensitivity at 33 μM as minimum peptide bactericidal concentration with about 25% increment in sensitivity indicative of higher antibacterial potency of this novel hybrid peptide compared to its counterpart single-peptide LL-37 as reported from our previous study with 64% sensitivity against *E. coli* and 69% against *S. aureus* (Perez-Perez et al. 2021). We also observed approximately a 2-fold reduction with respect to the minimum inhibitory hybrid peptide concentration required to inhibit bacterial growth as compared to its single-peptide LL-37. We envisioned that the hybrid peptide’s antimicrobial effects are brought about through its ability to disrupt cell membranes, thanks to its pronounced cationic polarity which enables it to create a strong electrostatic bond with the negatively charged bacteria membrane. Additionally, it is thought that the hybrid peptide’s v-shaped structure, made possible by the presence of the GS flexible linker, allows it to effectively engage with bacterial chromosomal DNA. This interaction can lead to the formation of supercoils, ultimately hindering DNA replication and transcription (Zhang et al. 2021).

In this study, we have designed, produced, and purified a novel multifunctional recombinant hybrid peptide LL-37_Renalexin for the first time via the application of newly designed flexible GS peptide linker and a characterized carrier proteins SmbP and CusF3H+. The small metal-binding protein tags SmbP and CusF3H+ provide an evidential advantage in cytoplasmic production and purification of the novel hybrid AMP LL-37_Renalexin with intact biochemical properties and can be applied as a new avenue to produce recombinant peptides and proteins. The purified tag-free hybrid peptide LL-37_Renalexin exhibited above 85% reduction in bacteria CFU/ml against *S. aureus*, *E. coli*, MRSA, and *K. pneumoniae* clinical isolates at lower minimum inhibition concentration levels of 10–33 μM as compared to its counterpart single-AMPs LL-37 and Renalexin of 50–100 μM reported (Aleinein et al. 2013; Kang et al. 2019; Perez-Perez et al. 2021), making it a competitive antimicrobial agent.

From our antibacterial bioassay findings, we proposed that the newly designed hybrid peptide LL-37_Renalexin can be classified as an antibacterial peptide that may have no toxic effects against normal cells at the niche of infection as theoretically predicted. Previous investigations (Aleinein et al. 2013; Jindal et al. 2015; Kang et al. 2019; Perez-Perez et al. 2021) have confirmed the exclusive antibacterial effect of recombinant single-peptides LL-37 and Renalexin, respectively, showing no toxic effects against normal human cells. Notably, we successfully express the hybrid peptide in *E. coli* with intact bioactivity conferred by the conserved secondary α-helical structures as seen in the single peptides supporting the predicted secondary structure of LL-37_Renalexin clearly showing the conserved helical domains of LL-37 and Renalexin. It is imperative to note, however, that this study lacks empirical data from wet laboratory circular dichroism (CD) spectrometry and 3-(4,5-dimthylthiazol-2-yl)-2,5-diphenyltetrazolium bromide (MTT) assay to support the theoretically predicted structure and toxicity. The recombinant DNA strategies used in the design, production, and purification of recombinant fusion proteins provide a reliable platform and protocol for the expression and purification of therapeutic recombinant proteins in *E. coli* BL21(DE3) and *E. coli* SHuffle T7(DE3) as microbial expression hosts.

## Supplementary Information

ESM 1 (PDF 2236 kb)

## Data Availability

All data supporting the findings of this study are available within the paper and its supplementary information.
