# Supplementary material for: LL-37_Renalexin hybrid peptide exhibits antimicrobial activity at lower MICs than its counterpart single peptides
Source: Appl Microbiol Biotechnol. 2024 Jan 13;108:126. doi: 10.1007/s00253-023-12887-5 (PMC10787891; doi:10.1007/s00253-023-12887-5)
Supplement: Supplementary file 1 [file 253_2023_12887_MOESM1_ESM.pdf]

Journal: Applied Microbiology and Biotechnology

LL-37_Renalexin hybrid peptide exhibits antimicrobial activity at lower MICs than its counterpart single peptides

Julius Kwesi Narh[1], Nestor G. Casillas-Vega[2], and Xristo Zarate[1,*]

[1] Facultad de Ciencias Quimicas, Universidad Autonoma de Nuevo Leon, Avenida Universidad s/n, Ciudad Universitaria, San Nicolas de los Garza, NL, 66455, Mexico

[2] Departamento de Patologia Clinica, Hospital Universitario Dr. Jose Eleuterio Gonzalez, Universidad Autonoma de Nuevo Leon, Monterrey, NL, 64460, Mexico

* Corresponding author: Email: xristo.zaratekl@uanl.edu.mx Tel: 8183294000 ext. 3444

(A)

CATATG

ATGCACCACCACCACCACCACGAGACCATGAGCGAGGCCCAGCCCCAGGTGATCAGCG
CCACCGGCGTGGTGAAGGGCATCGACCTGGAGAGCAAGAAGATCACCATCCACCACGA
CCCCATCGCCGCCGTGAACTGGCCCGAGATGACCATGAGGTTCACCATCACCCCCCAGA
CCAAGATGAGCGAGATCAAGACCGGCGACAAGGTGGCCTTCAACTTCGTGCAGCAGGG
CAACCTGAGCCTGCTGCAGGACATCAAGGTGAGCCAGGTACCGACGACGACGATAAAC
TGCTGGGTGATTTCTTTCGTAAAAGCAAAGAGAAAATCGGCAAAGAGTTCAAGCGCATC
GTGCAGCGTATCAAGGACTTCCTGCGTAATCTGGTTCCGCGTACGGAAAGCGGTAGCTT
TTTAGGTGGTCTGATTAAGATCGTGCCGGCAATGATTTGTGCTGTGACCAAAAAGTGC

TAACTCGAG

(B)

CATATG

TCAGGACATACAGCTCACGTAGATGAAGCAGTCAAACACGCTGAAGAAGCGGTGGCCC

ATGGCAAGGAGGGCCACACCGATCAACTGCTGGAACACGCGAAGGAGTCGTTGACCCA

TGCGAAAGCGGCGTCTGAAGCGGGTGGAAACACCCACGTTGGTCACGGCATTAAGCAT

CTCGAGGATGCAATCAAGCACGGCGAAGAGGGTCATGTTGGCGTTGCTACCAAGCACG

CGCAGGAGGCCATTGAACATTTGCGCGCATCCGAGCACAAAAGCCATGGTACCGACGA

CGACGATAAACTGCTGGGTGATTTCTTTCGTAAAAGCAAAGAGAAAATCGGCAAAGAG

TTCAAGCGCATCGTGCAGCGTATCAAGGACTTCCTGCGTAATCTGGTTCCGCGTACGGA

AAGCGGTAGCTTTTTAGGTGGTCTGATTAAGATCGTGCCGGCAATGATTTGTGCTGTGAC

CAAAAAGTGC **TAACTCGAG**

Figure S1: DNA nucleotide sequence of cDNA (DNA insert). (A) cDNA nucleotide sequence encoding for CusF3H+_LL-37_Renalexin fusion protein**.** (B) cDNA nucleotide sequence encoding or SmbP_LL-37_Renalexin fusion protein.

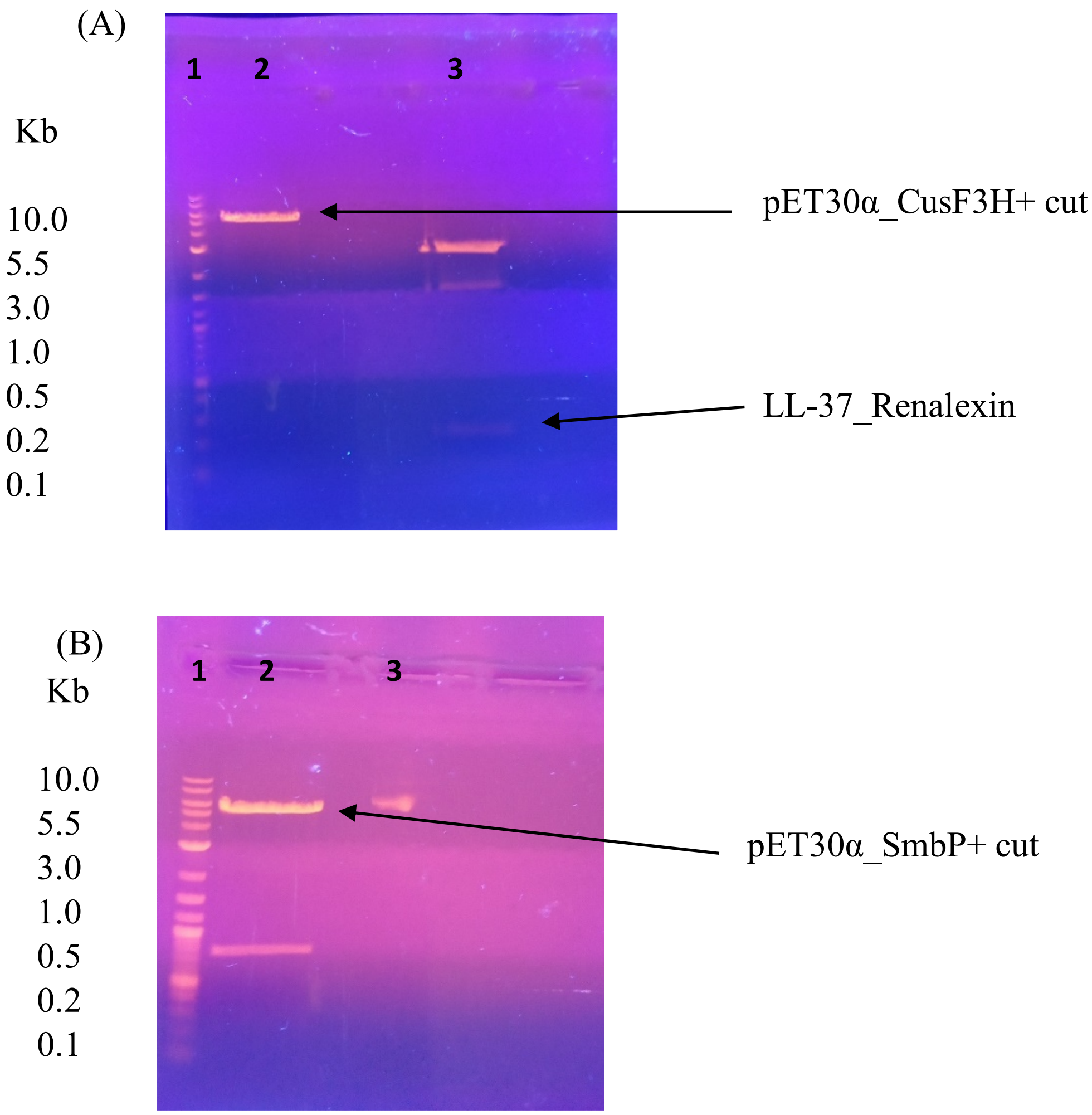

Figure S2: Agarose gel analysis of restriction enzyme digestion of plasmid DNA. (A) 1% Agarose gel analysis of restriction digestion: Lane1; DNA ladder, Lane 2: pET30α_CusF3H+ cut, Lane3; pUC57_SmbP_LL-37_Renalexin cut. (B): 1% Agarose gel analysis of restriction digestion: Lane1; DNA ladder, Lane2; pET30α_SmbP_GFP cut, Lane 3; pET30α_SmbP_GFP uncut.

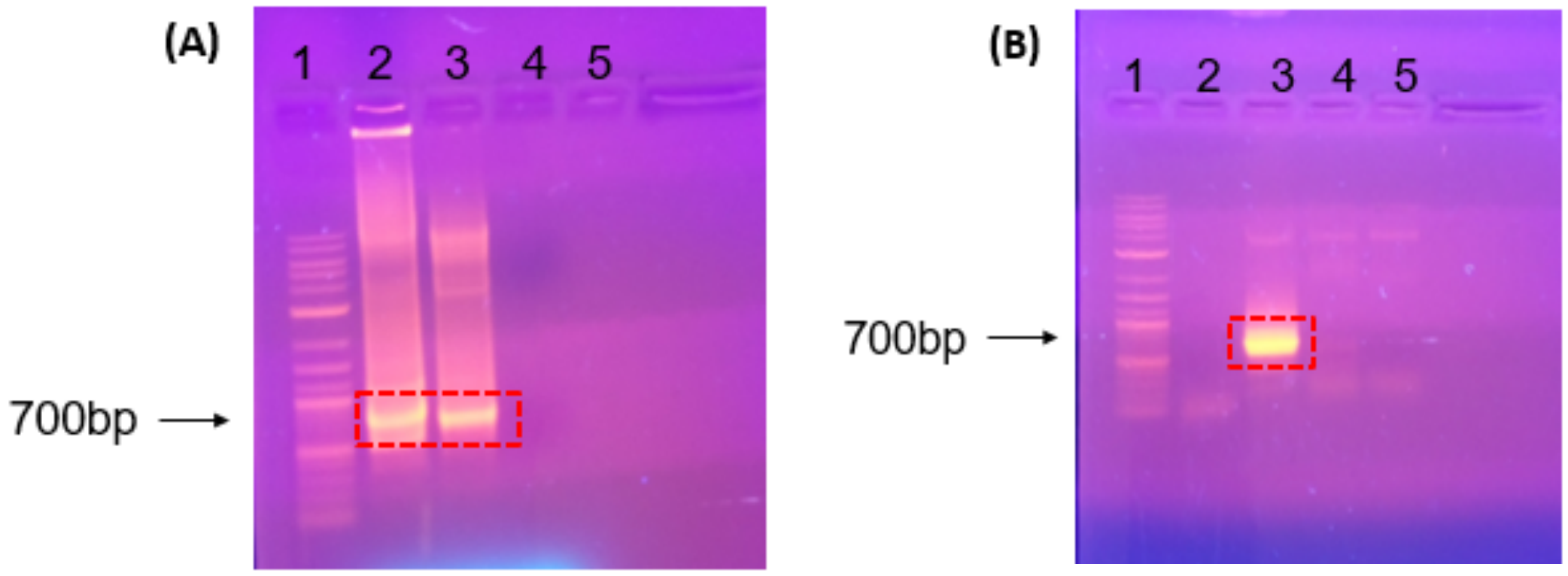

Figure S3: PCR analysis of synthetic DNA inserts in the designed recombinant plasmid constructs (A): 1% Agarose gel analysis of CusF3H+_LL-37_Renalexin DNA amplicons: Lane 1; DNA ladder, Lane 2, 3 & 4; PCR amplicons, Lane 5: Negative control (No DNA sample). Amplicons in the red rectangular label indicate the target amplifications. (B): 1% Agarose gel analysis of SmbP_LL-37_Renalexin DNA amplicons: Lane 1; DNA ladder, Lane 2; Negative control (No DNA sample), Lane: 3,4 & 5; PCR amplicons. Amplicons in the red rectangular label indicate the target amplifications.

(A)

```
# Length: 480
# Identity:     480/480 (100%)
# Similarity:   480/480 (100%)
# Gaps:           0/480 (0.0%)
# Score: 1662
#
#
#=======================================

EMBOSS_001        10 CACCACCACCATCATCATGAAACCATGAGCGAAGCACAACCACAGGTTAT     59
                     ||||||||||||||||||||||||||||||||||||||||||||||||||
EMBOSS_001        85 CACCACCACCATCATCATGAAACCATGAGCGAAGCACAACCACAGGTTAT    134

EMBOSS_001        60 TAGCGCCACTGGCGTGGTAAAGGGTATCGATCTGGAAAGCAAAAAAATCA    109
                     ||||||||||||||||||||||||||||||||||||||||||||||||||
EMBOSS_001       135 TAGCGCCACTGGCGTGGTAAAGGGTATCGATCTGGAAAGCAAAAAAATCA    184

EMBOSS_001       110 CCATCCATCACGATCCGATTGCTGCCGTGAACTGGCCGGAGATGACCATG    159
                     ||||||||||||||||||||||||||||||||||||||||||||||||||
EMBOSS_001       185 CCATCCATCACGATCCGATTGCTGCCGTGAACTGGCCGGAGATGACCATG    234

EMBOSS_001       160 CGCTTTACCATCACCCCGCAGACGAAAATGAGTGAAATTAAAACCGGCGA    209
                     ||||||||||||||||||||||||||||||||||||||||||||||||||
EMBOSS_001       235 CGCTTTACCATCACCCCGCAGACGAAAATGAGTGAAATTAAAACCGGCGA    284

EMBOSS_001       210 CAAAGTGGCGTTTAATTTTGTCCAGCAGGGCAACCTTTCTTTATTACAGG    259
                     ||||||||||||||||||||||||||||||||||||||||||||||||||
EMBOSS_001       285 CAAAGTGGCGTTTAATTTTGTCCAGCAGGGCAACCTTTCTTTATTACAGG    334

EMBOSS_001       260 ATATTAAAGTCAGCCAGGGTACCGACGAAAAGGATATACTGCTGATGGAT    309
                     ||||||||||||||||||||||||||||||||||||||||||||||||||
EMBOSS_001       335 ATATTAAAGAAAGCCAGGGTGCCGACCGGCAGGATCTACAACCGCTTGGT    384
                     ||||||||||||||||||||||||||||||||||||||||||||||||||
EMBOSS_001       310 ATATTAAAGAAAGCCAGGGTGCCGACCGGCAGGATCTACAACCGCTTGGT    359
```

(B)

```
# Length: 492
# Identity:     492/492 (100%)
# Similarity:   492/492 (100%)
# Gaps:           0/492 (0.0%)
# Score: 1831
#
#
#=======================================

EMBOSS_001         1 CATATGTGAGGACATACAGCTCACGTAGACGAAGCAGTGAAGCACGCTGA     50
                     ||||||||||||||||||||||||||||||||||||||||||||||||||
EMBOSS_001        31 CATATGTGAGGACATACAGCTCACGTGGACGAAGCAGTGAAGCACGCTGA     80

EMBOSS_001        51 AGAAGCGGTGGCCCATGGCAAAGAAGGCCATACCGATCAATTACTGGAAC    100
                     ||||||||||||||||||||||||||||||||||||||||||||||||||
EMBOSS_001        81 GGAAGCGGTGGCCCATGGTAAAGAAGGCCATACCGATCAATTACTGGAAC    130

EMBOSS_001       101 ATGCGAAGGAATCTCTGACACATGCGAAAGCTGCGACTGAAGCAGGTGGA    150
                     ||||||||||||||||||||||||||||||||||||||||||||||||||
EMBOSS_001       131 ATGCGAAGGAATCTCTGACACATGCGAAAGCTGCGACTGAAGCAGGTGGA    180

EMBOSS_001       151 AACACGCATGTTGGTCACGGAATCAAACATCTCGAAGATGCAATCAAGCA    200
                     ||||||||||||||||||||||||||||||||||||||||||||||||||
EMBOSS_001       181 AACACGCATGTTGGTCACGGAATCAAACATCTCGAAGATGCAATCAAGCA    230

EMBOSS_001       201 CGGCGAAGAGGGTCATGTCGGTGTTGCTACCAAGCATGCGCAAGAGGCTA    250
                     ||||||||||||||||||||||||||||||||||||||||||||||||||
EMBOSS_001       231 CGGCGAAGAAGGTCATGTCGGTGTTGCTACCAAGCATGCGCAAGAGGCTA    280

EMBOSS_001       251 TCGAGCATTTGCGTGCATCCGAACATAAATGGCACGGTACCGTCGACGTT    300
                     ||||||||||||||||||||||||||||||||||||||||||||||||||
EMBOSS_001       281 TCGAGCATTTGCGTGCATCCGAACATAAATGGCACGGTACCGTGGACGTT    330

EMBOSS_001       301 GAGCTAAACTGCTCGGAAATGTTCAATCGTAAACGCCTACACAAGCTTGG    347
                     ||||||||||||||||||||||||||||||||||||||||||||||||||
EMBOSS_001       331 GAGCTAAACTGCTCGGAAATGTTCAATCGTAAACGCCTACACGAGCTTGG    377
```

Figure S4: DNA nucleotide sequence alignment using the Needleman−Wunsch algorithm for pairwise analysis between the synthetic cDNA and designed plasmid DNA insert (amplicon). (A) Sequence nucleotide alignment for CusF3H+_LL-37_Renalexin DNA amplicon. (B) Sequence nucleotide alignment for SmbP_LL-37_Renalexin DNA amplicon.

Table S1: Protein (recombinant fusion peptides) band purity using densitometry in ImageJ.

Densitometry in ImageJ							
ID	Area	Mean	Min	Max	Max – Min	% Purity	
Lysate	446080	122.752	69	167	98		
Elution 1	446080	145.113	66	170	104	84.72367	
Elution 2	446080	145.912	66	165	99	80.65042	
Elution 3	446080	148.103	66	177	111	90.42623	
Elution 4	446080	149.279	64	182	118	96.12878	
Elution 5	446080	151.18	63	175	112	91.24088	
Elution 6	472320	150.977	66	180	114	92.87018	
Elution 7	472320	154.113	66	179	113	92.05553	

Determination of recombinant fusion peptide concentration using Bradford analysis with Bovine Serum Albumin (BSA) as standard protein

Table S2: CusF3H+_LL-37_Renalexin, expressed in *E. coli* BL21(DE3), quantification.

BSA (mg/ml)	Absorbance (595nm) A	B	C	average	ave-blnk
1.3	1.224	1.252	1.327	1.267667	0.648333
1	1.189	1.232	1.134	1.185	0.565667
0.7	1.084	1.062	1.045	1.063667	0.444333
0.5	0.963	0.99	0.936	0.963	0.343667
0.3	0.811	0.795	0.762	0.789333	0.17
0.1	0.694	0.669	0.65	0.671	0.051667
Sample	0.767	0.776	0.74	0.761	0.141667
Blank	0.644	0.612	0.602	0.619333	

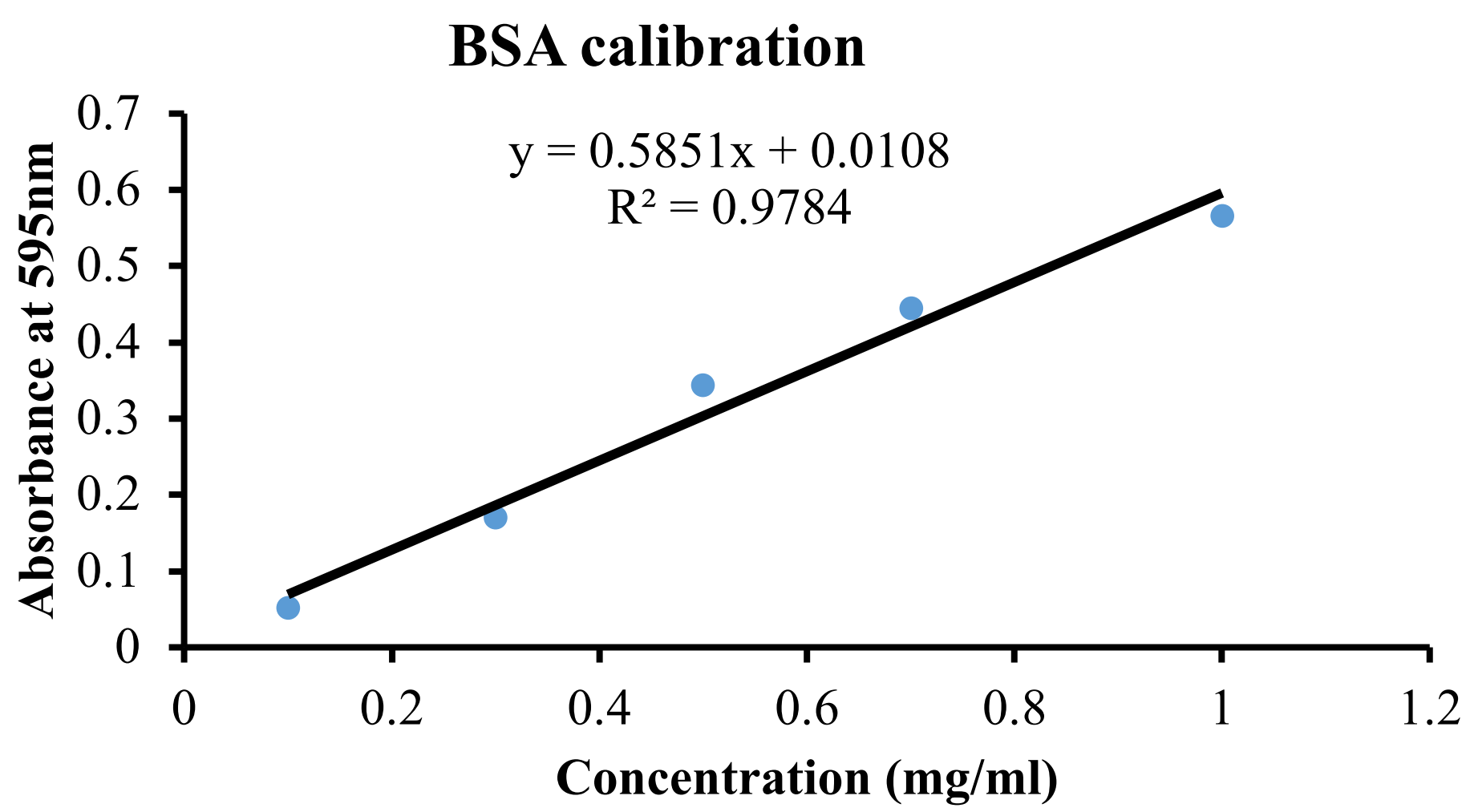

Figure S5: Calibration curve for CusF3H+_LL-37_Renalexin (with disulfide bond) quantification.

Concentration: (F9-0.0108)/0.5851= **0.223665mg/ml X** total elution fractions (14) =**3.136mg/L**

F9= 0.141667.

Table S3: SmbP_LL-37_Renalexin, expressed in *E. coli* BL21(DE3), quantification.

BSA (mg/ml)	Absorbance (595nm) A	Abs B	Abs C	Average Abs	Av Abs – blnk
1	0.989	1.042	1.057	1.029333	0.450666333
0.7	0.969	0.92	0.91	0.933	0.354333
0.5	0.86	0.836	0.839	0.845	0.266333
0.3	0.698	0.676	0.687	0.687	0.108333
0.2	0.697	0.756	0.687	0.713333	0.134666333
0.1	0.716	0.615	0.614	0.648333	0.069666333
Sample	0.654	0.662	0.639	0.651667	0.072999667
Blank	0.546	0.589	0.601	0.578667	

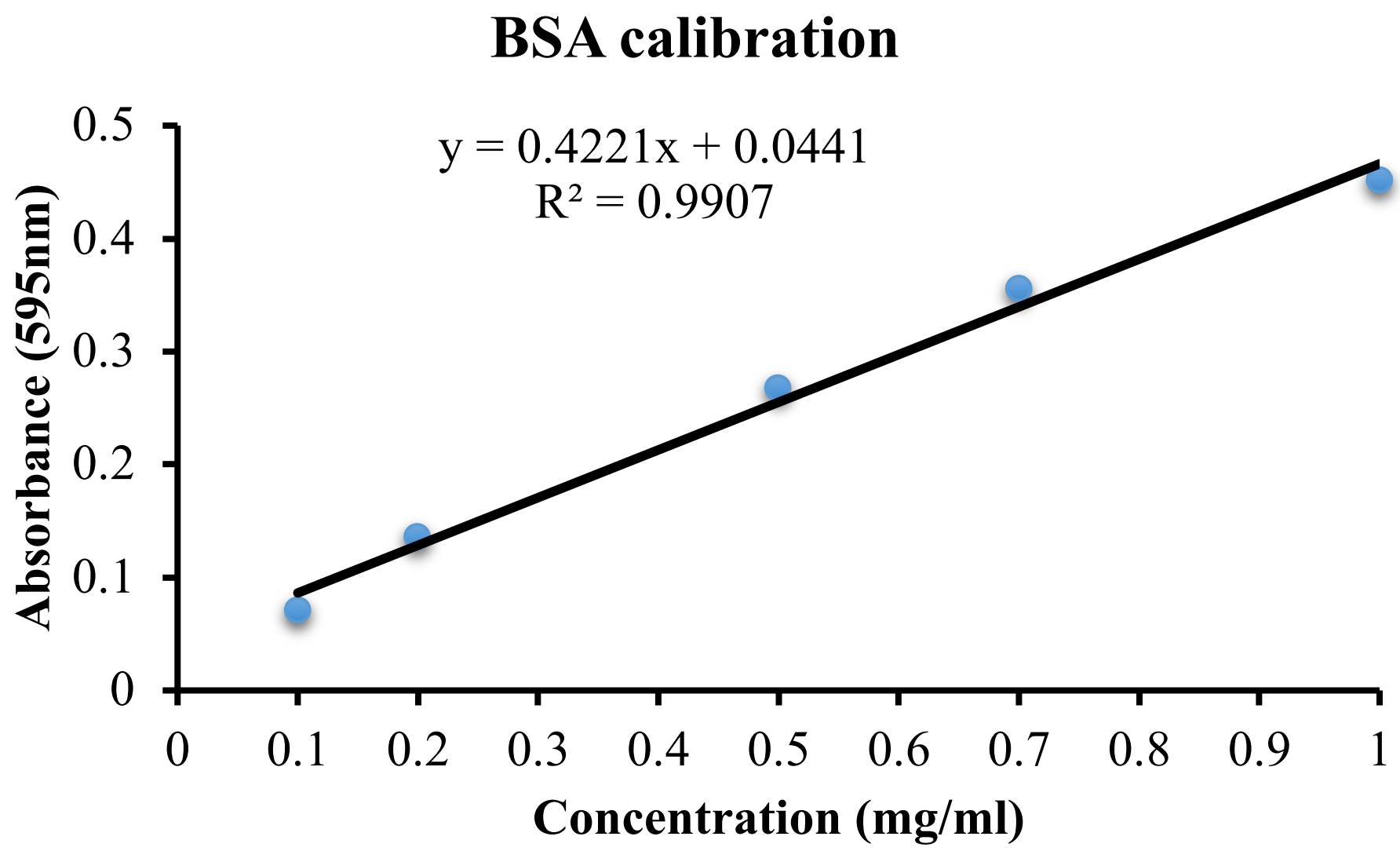

Figure S6: Calibration curve for SmbP_LL-37_Renalexin (without disulfide bond) quantification.

Concentration= (F9-0.04221)/0.4221 = **0.1mg/ml** X total elutions (15) = **1.5mg/L**

F9= 0.07299967.

Concentration after enterokinase cleavage

Table S4: LL-37_Renalexin (expressed in *E. coli* SHuffle T7) cleaved, quantification using NanoDrop at (A_{280nm})

BSA (mg/ml)	A	B	C	Ave Abs
1.3	0.97	0.997	1.001	0.989333
1	0.75	0.749	0.747	0.748667
0.7	0.487	0.512	0.507	0.502
0.5	0.341	0.359	0.356	0.352
0.3	0.22	0.193	0.201	0.204667
0.1	0.084	0.061	0.103	0.082667
Sample	0.105	0.0956	0.0958	0.0988
Blank	0	0	0	0

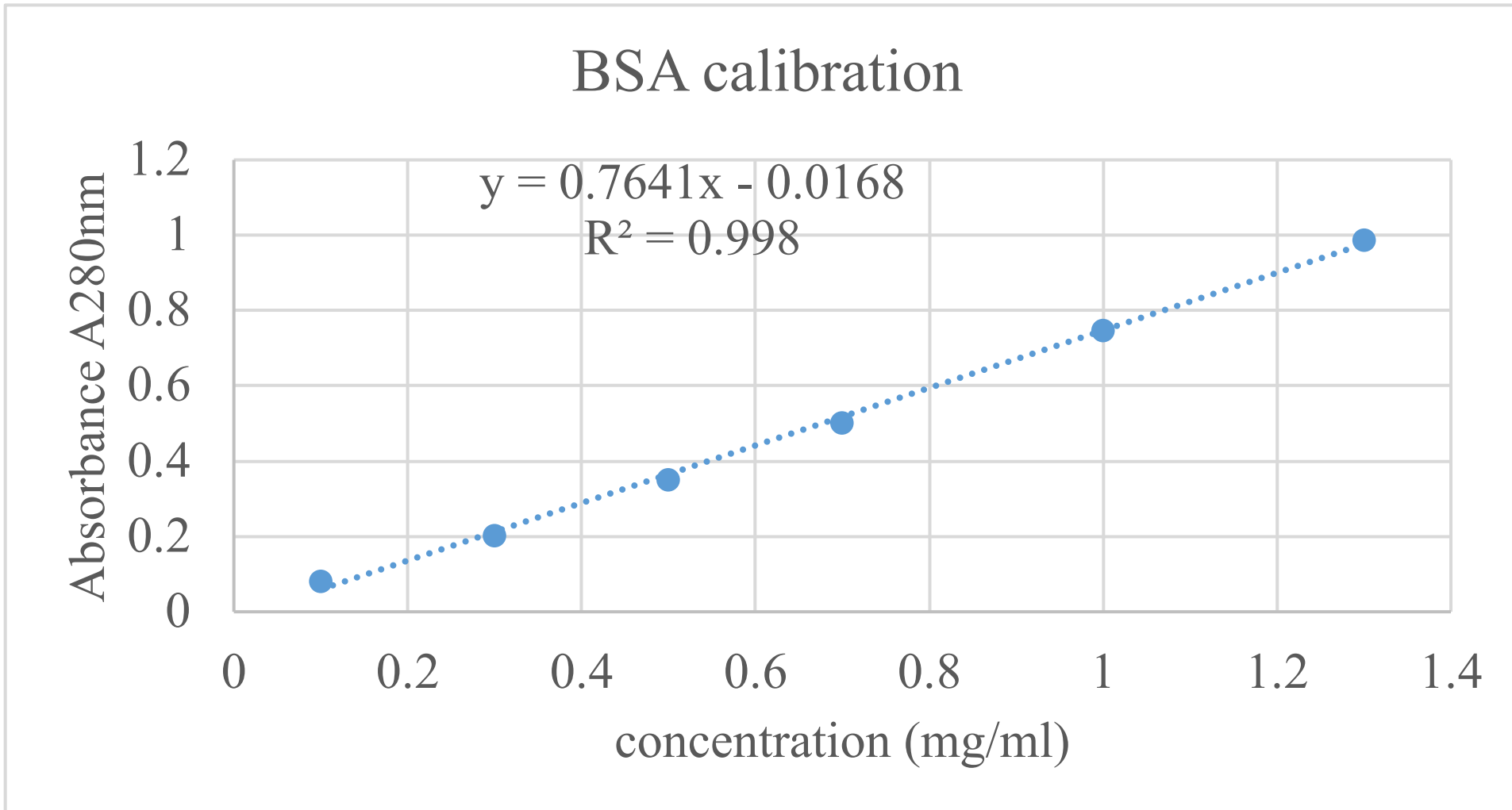

Figure S7: Calibration curve for LL-37_Renalexin (with disulfide bond) cleaved.

Concentration= (F9+0.0168)/0.7641 = **0.151mg/ml** X total elutions (14) = **2.1mg/L**

F9= 0.0988

Table S5: LL-37_Renalexin, expressed in *E. coli* BL21(DE3), cleaved, quantification using NanoDrop at (A_{280nm}).

BSA	A	B	C	Average
1.3	0.956	0.955	0.952	0.954333
1	0.712	0.701	0.717	0.71
0.7	0.464	0.494	0.495	0.484333
0.5	0.333	0.34	0.342	0.338333
0.3	0.175	0.228	0.183	0.195333
0.1	0.072	0.073	0.086	0.077
Sample	0.02	0.019	0.013	0.017333

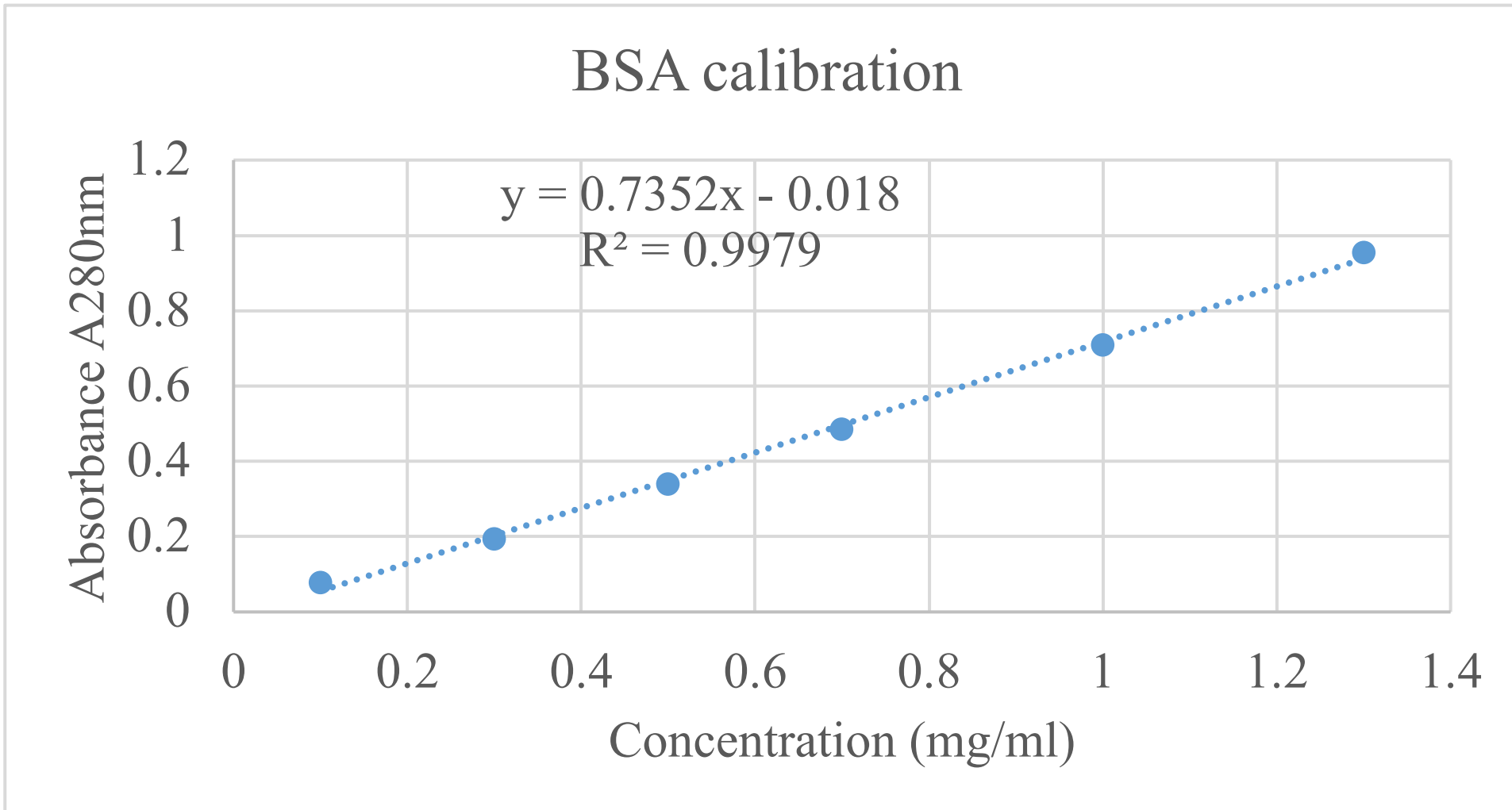

Figure S8: Calibration curve for LL-37_Renalexin (without disulfide bond) cleaved.

Concentration= (F9+0.018)/0.7352 = **0.0467mg/ml** X total elutions (15) = **0.7mg/L**

F9= 0.01733

Antimicrobial activity (Dose_response) assay

Table S6: LL-37_Renalexin (expressed in *E. coli* SHuffle T7) against *Staphylococcus aureus*

Peptide concentration (µM)	CFU/plate					
	Rep1	Rep2	Rep3	average	Reduction	% Reduction
0	81	70	64	72	0	0
0.5	51	65	60	59	13	13
1	49	60	57	55	17	19
2.5	45	52	52	50	22	26
5	44	38	46	43	29	37
10	35	28	40	34	38	50
15	25	14	22	20	52	71
20	17	14	9	13	59	81
25	12	11	9	11	61	84

30	9	11	7	9	63	87
33	6	9	7	7	65	90

Table S7: LL-37_Renalexin (expressed in *E. coli* SHuffle T7) against *Escherichia coli*

Conc (µM)	CFU/Plate					
	Rep1	Rep2	Rep3	Average	Reduction	% Reduction
0	101	126	113	113	0	0
0.5	97	89	94	93	20	18
1	84	80	79	81	32	29
2.5	82	72	80	78	35	31
5	69	61	70	67	47	41
10	54	60	58	57	56	49
15	52	50	47	50	64	56
20	37	40	31	36	77	68
25	32	25	21	26	87	77
30	24	15	19	19	94	83
33	13	14	17	15	99	87

Table S8: LL-37_Renalexin, expressed in *E. coli* BL21(DE3), against *Staphylococcus aureus*

Peptide concentration (µM)	CFU/plate					
	Rep1	Rep2	Rep3	Average	Reduction	% Reduction
0.5	71	69	81	74	0	0
1	69	68	74	70	3	5
1.5	64	67	71	67	6	9
3	63	62	70	65	9	12
6	47	54	50	50	23	32
8	41	35	39	38	35	48
10	27	35	33	32	42	57
12	23	26	30	26	47	64
15	19	25	20	21	52	71
20	13	11	17	14	60	81
25	12	10	16	13	61	83

Table S9: LL-37_Renalexin, expressed in *E. coli* BL21(DE3), against *Escherichia coli*

Peptide concentration (µM)	CFU/plate					
	Rep1	Rep2	Rep3	Average	Reduction	% Reduction
0	102	98	111	104	0	0
0.5	86	90	80	85	18	18
1	80	88	81	83	21	20
1.5	80	70	78	76	28	27
3	74	68	70	71	33	32
6	71	67	62	67	37	36
8	70	66	60	65	38	37
10	65	64	60	63	41	39
12	61	59	58	59	44	43
15	56	54	51	54	50	48
20	49	46	43	46	58	56
25	35	29	31	32	72	69

Antimicrobial activity (Time-killing kinetic assay)

Table S10: LL-37_Renalexin (expressed in *E. coli* SHuffle T7) against *Staphylococcus aureus*

Time (h)	CFU/ml								
	LL-37_Renalexin (33µM)			Kanamycin (33µM)			Control		
0	41	45	37	43	37	40	47	39	43
0.3	45	47	43	28	22	25	49	51	50
0.6	56	50	62	18	22	20	65	59	62
1	49	45	51	15	11	13	67	61	64
1.3	57	55	59	11	7	9	83	75	79
1.6	40	37	43	5	9	7	84	80	82
2	31	25	37	5	3	4	88	84	86
2.3	26	29	23	3	3	3	90	94	92
2.6	21	19	23	1	5	3	92	96	94
3	11	10	12	2	4	3	96	98	97

Table S11: LL-37_Renalexin (expressed in *E. coli* SHuffle T7) against *Escherichia coli*

Time (h)	LL-37_Renalexin (33µM)			Kanamycin (33µM)			Control		
0	57	55	57	55	51	50	61	57	51
0.3	58	49	59	29	31	34	68	59	66
0.6	63	62	59	19	21	23	79	83	69
1	65	61	55	11	14	15	85	78	81
1.3	49	50	40	7	9	7	87	97	89
1.6	25	21	27	4	7	5	117	109	122
2	15	17	14	3	2	2	119	121	130
2.3	12	10	11	1	2	0	132	140	143
2.6	9	7	11	0	0	0	145	147	150
3	4	5	6	0	0	0	150	151	149

ANOVA (With total possible CFU/ml)

Table S12: LL-37_Renalexin (expressed in *E. coli* SHuffle T7) against *Staphylococcus aureus*

Average of CFU/ml (3Reps)		
Control	LL-37_Renalexin (33µM)	Kanamycin (33µM)
43	41	40
50	45	25
62	56	20
64	49	13
79	57	9
82	40	7
86	31	4
92	26	3
94	21	3
97	11	3

Table S13: LL-37_Renalexin (with disulfide bond) against *Escherichia coli*

Average CFU/ml (3Reps)		
Control	LL-37_Renalexin (33µM)	Kanamycin (33µM)
61	57	55

68	58	29
79	63	19
85	65	11
87	49	7
117	25	4
119	15	3
132	12	1
145	9	0
150	4	0

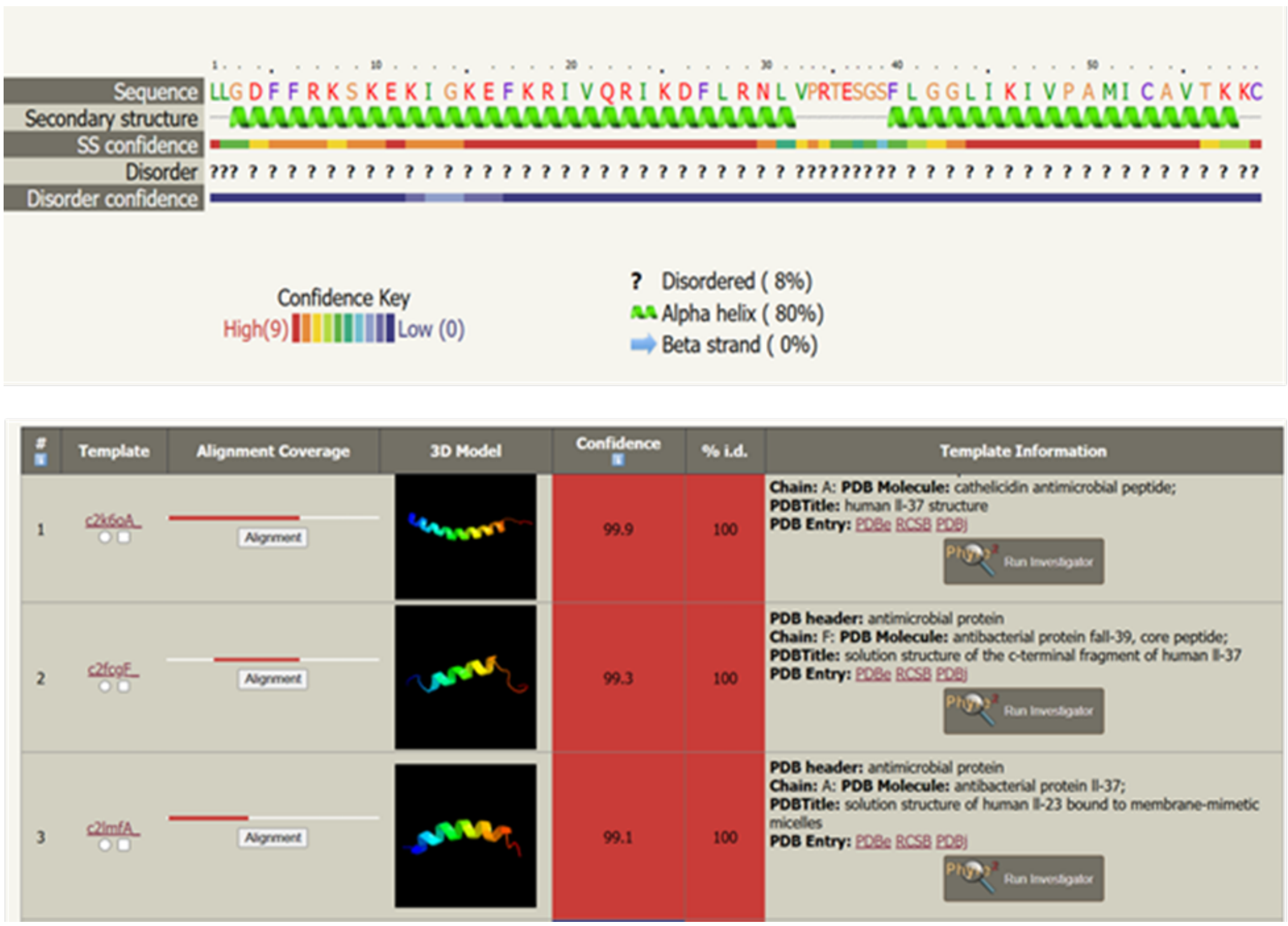

Figure S9: In-silico confirmation of the i-Tasser predicted secondary structure of LL-37_Renalexin using the Phyer2 server (http://www.sbg.bio.ic.ac.uk/phyre2). The amino acid sequence of the hybrid peptide was submitted to the server with all parameters under default.

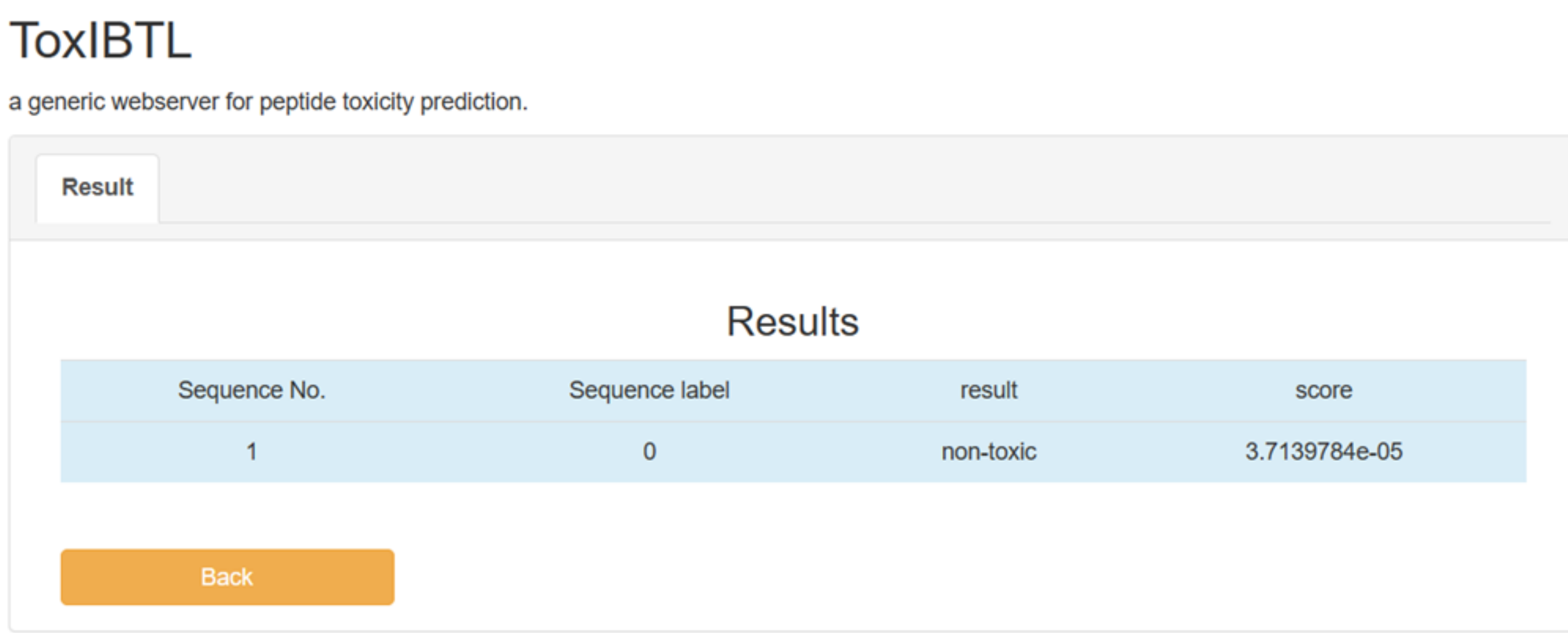

Figure S10: In-silico cellular toxicity against normal human cells prediction of the hybrid peptide (LL-37_Renlexin) in the ToxIBTL server. The amino acid sequence of the hybrid peptide was submitted to the server in FASTA format. The predicted toxicity score was 3.7139784e-05 (0.0000371), which was far below the threshold of 0.5, indicating a zero-peptide toxic effect on healthy human cells.

Table S14: LL-37_Renalexin expressed in *E. coli* SHuffle T7(DE3) against MRSA

Conc (uM)	Colony Forming Units		
	Rep 1	Rep 2	Rep 3
0	101	119	108
0.5	87	89	78
1	65	70	76
2.5	58	63	69
5	68	57	59
10	49	56	50
15	46	51	45
20	42	33	38
25	19	29	32
30	13	12	14
35	11	13	10

Table S15: LL-37_Renalexin expressed in *E. coli* SHuffle T7(DE3) against *K. pneumonaie*

Conc (uM)	Colony Forming Units		
	Rep 1	Rep 2	Rep 3
0	101	120	108
0.5	90	98	91
1	80	82	79
2.5	77	69	74
5	66	65	70
10	54	61	59
15	50	52	48
20	43	41	44
25	33	19	25
30	24	10	15
35	16	10	11
